# Advances in Functional Hydrogel Wound Dressings: A Review

**DOI:** 10.3390/polym15092000

**Published:** 2023-04-23

**Authors:** Zihao Shen, Chenrui Zhang, Ting Wang, Juan Xu

**Affiliations:** 1 Aulin College, Northeast Forestry University, Harbin 150040, China; zihaoshenhugh@163.com (Z.S.); BarbaraZhang2000@aliyun.com (C.Z.); 2 College of Chemistry, Chemical Engineering and Resource Utilization, Northeast Forestry University, 26 Hexing Road, Harbin 150040, China; 3 National Research Institute for Family Planning, Haidian District, No. 12, Da Hui Si Road, Beijing 100081, China

**Keywords:** hydrogel dressing, functionality, wound repair, biological materials

## Abstract

One of the most advanced, promising, and commercially viable research issues in the world of hydrogel dressing is gaining functionality to achieve improved therapeutic impact or even intelligent wound repair. In addition to the merits of ordinary hydrogel dressings, functional hydrogel dressings can adjust their chemical/physical properties to satisfy different wound types, carry out the corresponding reactions to actively create a healing environment conducive to wound repair, and can also control drug release to provide a long-lasting benefit. Although a lot of in-depth research has been conducted over the last few decades, very few studies have been properly summarized. In order to give researchers a basic blueprint for designing functional hydrogel dressings and to motivate them to develop ever-more intelligent wound dressings, we summarized the development of functional hydrogel dressings in recent years, as well as the current situation and future trends, in light of their preparation mechanisms and functional effects.

## 1. Introduction

**Figure 1:**
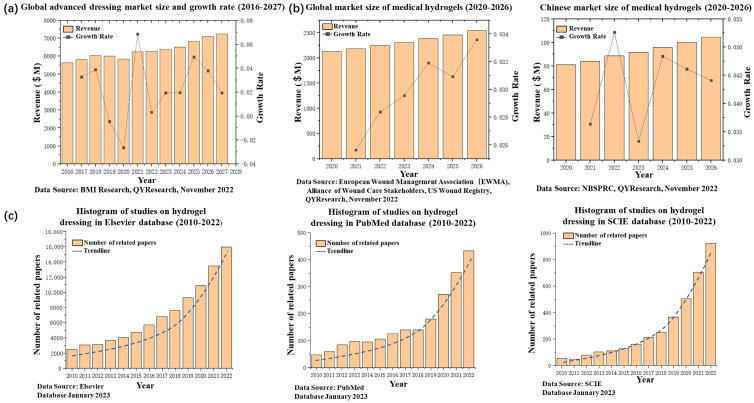
(**a**) Statistics and forecast of global high-end dressing market sales and growth rate (2016–2027). (**b**) Global market size of medical hydrogels and Chinese market size of medical hydrogels. (2020–2026). (**c**) Number of papers published per year related to hydrogel dressings in different databases (2010–2022).

In the field of global clinical medicine, the development of excellent wound dressings has become a central issue in the important challenge of wound repair [1]. It has also been a long-lasting research hotspot with great interest in a range of medicine and chemistry fields. According to the data from QY Research [2], it is predicted that the global advanced wound dressing market will increase from USD 6267 million in 2022 to USD 7230 million in 2027 (Figure 1a). The last two decades have seen a growing trend towards a variety of advanced wound dressings, such as alginate dressings [3], polymeric film dressings [4], hydrocolloid dressings [5], and hydrogel dressings [6]. Of particular concern, hydrogel wound dressings are considered to be the most competitive advanced wound dressing material [7]. This is because, on the one hand, hydrogel wound dressings have created a big medical market [8]. Figure 1b reveals that there has been a steady rise in the market demand [9] of medical hydrogels globally and in China since 2020. On the other hand, as shown in Figure 1c, a number of published papers on hydrogel dressings in different databases have all shown an exponential growth trend in the past decade (data source: Elsevier Database, PubMed Database, SCIE Database; 2023). Therefore, hydrogel wound dressings that can promote wound healing have the benefit of long-term, in-depth research to help them become advance dressings, and their composition and preparation mechanisms are the keys to their therapeutic effectiveness.

The main components of hydrogel wound dressings are 3D macromolecular networks and water [10]. In 1962, Winter et al. published a paper describing how skin cells regenerate faster in a medium-humidity environment [11]. This was the first study to thoroughly examine the theory of wet healing. According to this theory, hydrogel dressings can not only help accelerate wound healing [12] but can also help patients alleviate aches by cooling the wound [13]. Furthermore, as a wound dressing, hydrogel can absorb excess tissue exudates while also providing good oxygen permeability [14]. These advantages are too obvious to ignore as they create condition for a potentially ideal wound dressing. However, traditional hydrogel wound dressings’ ability to become more advanced has been severely constrained by its poor mechanical performance, inability to avoid wound infection, and low wound healing efficiency [15]. The best solution to these problems is to develop hydrogel dressings with more functions, which is inevitable and crucial.

The role of hydrogel dressings is changing from physical coverage to “functionalization” as more attention is being paid to functional hydrogel dressings [16]. The urge to develop a quick response to different wound conditions to improve the healing effect has been the driving force towards the “functionalization” of hydrogel dressings, including the application of novel materials and various advanced preparation mechanisms [17]. Most of functional hydrogel dressings can play vital roles in some or all of the three stages of wound healing: hemostasis/inflammation, proliferation and remodeling [18,19].

**Figure 2:**
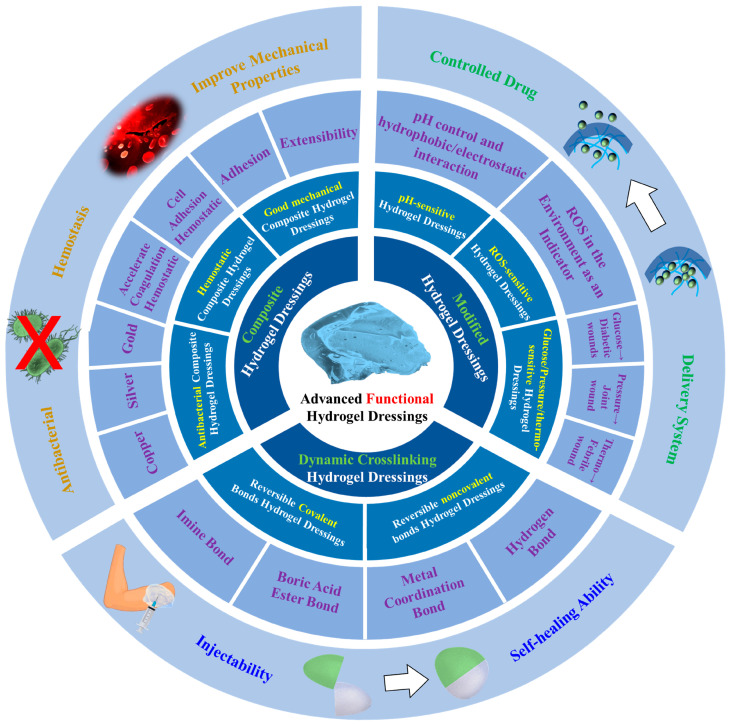
Contents and applications of this review.

As shown in Figure 2, hydrogel dressings’ self-healing and injectable functions allow them to completely cover the surface of a wound. The selection of covalent [20] and non-covalent [21] bonds as the base interactions for the formation of dynamic crosslinking hydrogels should not only be considered as an effective approach for both injectability and self-healing applications, but also as affecting the healing efficiency. Currently, hydrogel dressings with self-healing and injectable functions [22] have the ability of immediate reorganization and integration and the ability to mold to different shapes of wounds within a few seconds [23].

Hydrogel dressings can also carry various functional drugs and deliver them directly to the wound site [24]. In order to obtain this function, the release of the functional drugs by the hydrogel dressings must be designed and applied according to different wound microenvironments and use specific signals, such as pH, ROS, glucose, pressure, and temperature, to control functional drug release, thus greatly promoting the speed and effect of wound healing [25].

Furthermore, hydrogel dressings can possess a range of functions and properties, including antibacterial activity, hemostatic ability, and mechanical strength, depending on the specific design and composition [26]. Chemical particles with strong antibacterial properties can be utilized for sterilization or bacteriostasis in the vicinity of the wound. However, it is important to carefully assess the potential negative effects of these chemical particles on human health [27,28,29]. Rapid and successful hemostasis can prevent shock or life-threatening bleeding when the wound is experiencing continuous blood flow. At present, functional hydrogel dressings prepared by loading chemical particles have realized the excellent hemostatic function of blood coagulation in tens of seconds [30]. In terms of mechanical properties, the adhesion and tensile properties of hydrogel dressings can be improved. For adhesion [27], the surface bonding or physical actions that can improve the chemical reaction between chemical particles and other substances can be used to achieve reasonable changes in adhesion. To improve extensibility [31], the diversity of evenly dispersed chemical particles and cross-linking points in the hydrogel can be used to improve the mechanical toughness. Improving the mechanical properties of the hydrogel can ensure that the dressing does not easily to fall off and that it will not cause secondary injury in the clinical application process.

Therefore, considering the three most typical functional hydrogel dressings mentioned above, we must innovate and design new chemicals and manufacturing methods to further improve their functionality [32].

Herein, this paper aims to unravel the latest progress in functional hydrogel dressings surrounding their connection mechanisms, composite structures, chemical modification, and compositions. There are many studies on functional hydrogel dressings at the moment, but there are few papers that provide a systematic summary of the literature. Thus, the importance of this review is to explore various research ideas from different preparation mechanisms couple with their corresponding functionality, which will provide an important reference for researchers to design novel medical hydrogel dressings with properties such as injectability, self-healing, controlled drug release, healing promotion, antibacterial activity, hemostasis, improved mechanical properties, and so on. The information in this review also has a number of important implications for future research directions in the preparation of functional hydrogel dressings.

## 2. Dynamic Crosslinking Hydrogel Dressings

**Figure 3:**
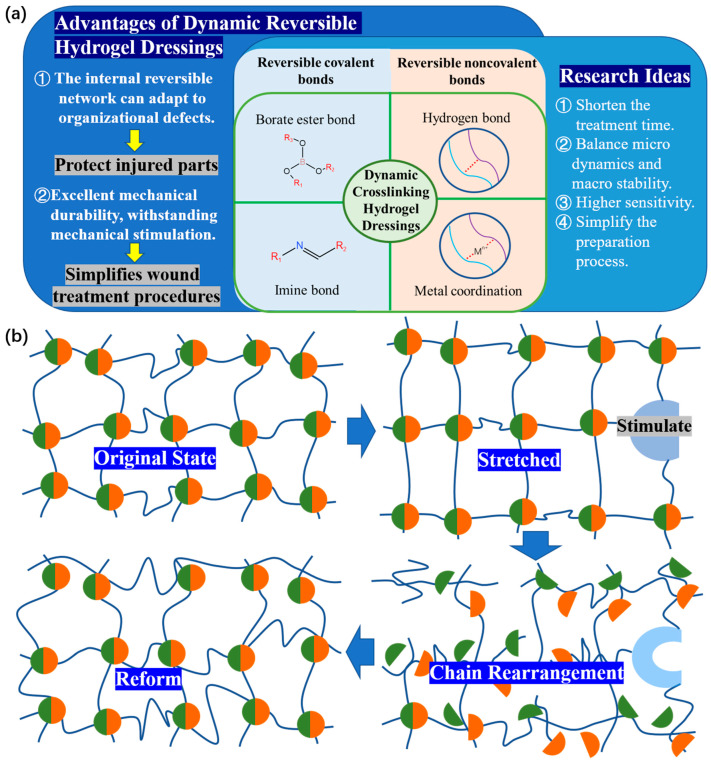
(**a**) The research ideas and (**b**) principles of self-healing and injectability of dynamic reversible hydrogel dressings.

Traditional dressings lack the benefits of self-healing and injectability during the clinical process [33]. Due to the strong rigidity and nondynamic nature of the skeleton, hydrogel is prone to deformation or damage caused by external mechanical forces, resulting in a shortened service life and making it difficult to maintain full contact with the wound, especially near joints, resulting in an unsatisfactory treatment effect [34,35]. For example, traditional dressings attached to fingers do not fit to finger wounds well and easily deform and fall off due to the frequent bending of the fingers [36]. Injectable hydrogels can fill irregular wound areas and promote in situ tissue regeneration, and self-healing hydrogels can withstand external mechanical forces, thus extending their service life [37]. So, to promote a favorable environment for wound repair, functional hydrogel dressings should have improved resistance to mechanical injury and debridement capability [38] (Figure 3a).

Hydrogels with self-healing and injectable properties can be prepared [39] by linking together through noncovalent cross-linking or dynamic covalent cross-linking (Figure 3b). According to the research and development findings of dynamic reversible hydrogel dressings, Zhang’s team proposed a few research ideas for the further development of dynamic hydrogels [40]: ① It is necessary to reduce the time required for the dynamic application of wound treatment. ② It is necessary to achieve a better balance between micro-level dynamics and macro-level stability. ③ It is necessary to obtain higher sensitivity to characterize the dynamic characteristics of hydrogels in real time. ④ It is necessary to simplify the hydrogel preparation process and promote the wide application of dynamic hydrogels in basic research and clinical transformation. These ideas indicate direction for the further development of functional dynamic reversible gel dressings and wound repair.

The balance between the macro-level stability and micro-level dynamics of hydrogel dressings and the dressings’ application requirements should be taken into consideration when discussing the general expectations and design principles of the performance of dynamic hydrogel dressings. In practice, the most common reversible covalent bonds are imine bonds. There are also boric acid ester bonds, special dynamic covalent bonds that can undergo bond exchange reactions in a few seconds at room temperature [41]. Metal coordinate bonds and hydrogen bonds are the two most typical examples of reversible noncovalent bonds. This will serve as a starting point in this section to introduce the current success of research in this field.

### 2.1. Reversible Covalent Bonds

Reversible covalent bonds are typically reversible, meaning that bond exchanges can occur. According to numerous studies, hydrogel dressings can be restored through dynamic reversible covalent bonds [42], demonstrating their potential for self-healing and injectability.

#### 2.1.1. Imine Bonds

Since Schiff successfully prepared an imine bond for the first time in 1864, researchers have deeply studied the imine bond and lots of research experience has been accumulated [43]. Imine bonds have a dynamic reversible property. The dynamic reversible covalent bonds between these functional groups are the dominant driving force for the self-healing of hydrogels, as opposed to the simple physical adhesion among the broken hydrogel interfaces [44]. Hydrogel dressings dynamically crosslinked by imine bonds have excellent injectability and efficient self-healing ability [45].

**Figure 4:**
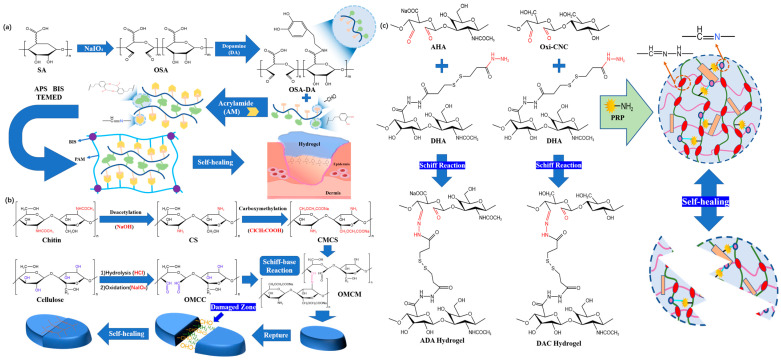
(**a**) The synthesis process and structural diagram of OSA-DA-PAM hydrogel. (**b**) The preparation of OMCM hydrogel based on Schiff base reactions and the scheme of the self-healing process. (**c**) AHA/DHA/oxi CNC hydrogel schematic diagram.

In earlier studies, Chen et al. [46] used dopamine-grafted oxidized sodium alginate (OSA-DA), polyacrylamide (PAM), and dopamine hydrochloride (DA) as the main raw materials to prepare OSA-DA-PAM hydrogel dressings. The hydrogel dressing was synthesized by dynamically covalently crosslinking the base reaction formed between the aldehyde group of the OSA-DA chain and the amino group of the PAM chain (Figure 4a). The resulting hydrogel can effectively self-heal without any external stimulation. However, the main weakness of this study is its failure to address injectability. Other preparation methods that are excellent in terms of both self-healing and injectability need to be found.

In view of the problems encountered in previous studies, Yin et al. [47] published a paper in which they described hydrogel dressings constructed using the aldehyde group of oxidized microcrystalline cellulose (OMCC) from pineapple peel (PP) crosslinked with the amino group of carboxymethyl chitosan (CMCS) from *hericium erinaceus residues* (HER) and using the Schiff base reaction for its construction (Figure 4b). Due to the continuous fracturing and regeneration of the imine bond, the researchers divided the network composed of the hydrogel dressing into two parts, and it took 5 h to achieve self-healing. For the requirements of wound dressings, the self-healing time of this product is too long. Dynamic covalent crosslinking can be achieved in hydrogel dressings through the use of specific products or components that facilitate the formation and exchange of reversible covalent bonds. These dynamic covalent crosslinking interactions contribute to the self-healing and injectable properties of hydrogel dressings. On the other hand, covalently crosslinked hydrogel dressings are not blocked during the injection process, showing good injection performance. These two characteristics aid in reducing pain and hastening the healing process for patients [48]. Similarly, by using imine bonds, Li et al. [49] generated self-healing hydrogel dressings composed of aldehyde-modified sodium hyaluronate (AHA), hydrazine-modified sodium hyaluronate (ADA), and aldehyde modified cellulose nanocrystals (oxi-CNC) (Figure 4c). When these hydrogel dressings are separated into two pieces, they can self-heal and rejoin into one cohesive piece after approximately 4 h. However, the self-healing times of the two items are still not optimal.

**Figure 5:**
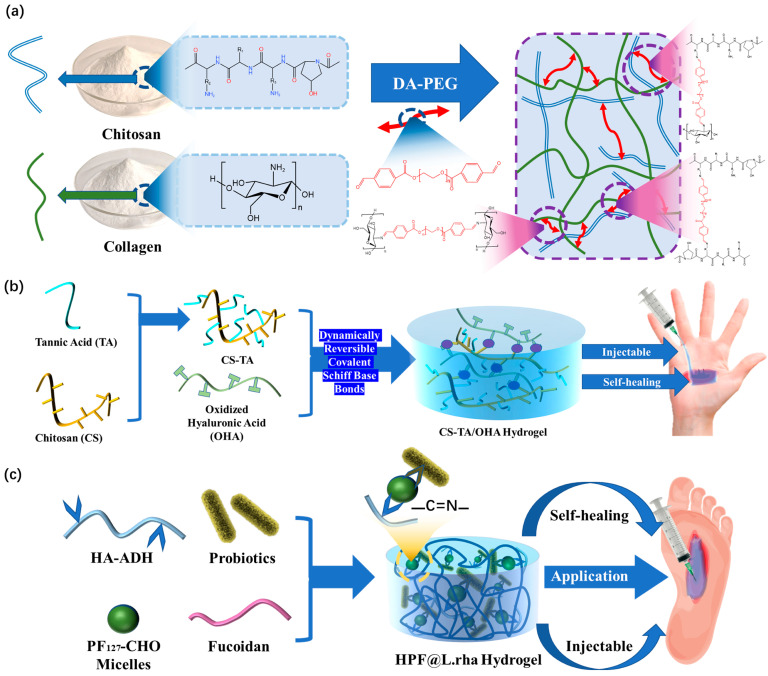
(**a**) Construction mechanism of COL-CS hydrogel. (**b**) The construction mechanism and functional mechanism of injectable and self-healing HPF@L.rha hydrogel dressings. (**c**) Design strategy and application method of CS-TA/OHA composite hydrogel dressings.

In order to improve the injectable ability and self-healing ability of hydrogel dressings, Ding et al. [50] demonstrated that collagen(CoL)–chitosan(CS) complex hydrogel can be applied to multi-functional wound dressings in the medical field. In their work, dibenzaldehyde-modified PEG_2000_(DA-PEG), a long-range crosslinker, was introduced in the mixed COL-CS network and was used as a dynamic crosslinker (Figure 5a). These characteristics aid in dynamically decoupling and recoupling imine connection, resulting in a superior self-healing hydrogel dressing. Functional hydrogel dressings’ self-healing times can be significantly decreased with the help of this preparation technique while still retaining good injectability. This is the critical stage for applying imine bonds in hydrogel dressings.

Previous research on the injectability and self-healing ability of hydrogels did not address the restoration of the original hydrogel’s mechanical properties. Exceptional performance of both properties is not only necessary to integrate the hydrogel dressing into a whole, but also to restore its excellent mechanical properties, such as its good tensile properties [51]. In order to achieve leapfrogging improvements, Mei et al. [52] modified hyaluronic acid (HA) with adipic hydrazide through acetylation to synthesize hyaluronic acid adipic hydrazide (HA-ADH). (Pluronic F_127_)-CHO and HA-ADH form dynamic covalent cross-links through Schiff base reaction interactions in the interventricular or physiological conditions (Figure 5b). The formation of the dynamic imine connection is what gives the dressing its self-healing property. It can bear high stress and can easily reform at low strain levels; meanwhile, the self-healing time is significantly reduced. In addition, other studies have further improved the performance of hydrogel dressings constructed by imine bonds. Using the dynamic reversible characteristics of Schiff base bonds between chitosan (CS), tannic acid (TA), and oxidized hyaluronic acid (OHA) [53], Liu’s hydrogel dressings were able to achieve traceless repair in a rather short time, which has a very short self-healing time compared to recent reports (Figure 5c). This excellent treatment time is a noteworthy achievement for dynamic reversible gel dressings prepared by imine bonds. It quickly achieves untraceable repair and has good injectability.

#### 2.1.2. Boric Acid Ester Bonds

Boric acid ester bonds are a special type of dynamic covalent bond that may change dynamically and reversibly at room temperature [54]. The bond exchange and repair times are also substantially shorter than those for imine bonds [55], which enhances their capacity for self-healing.

This hydrogel material is based on a dynamically reversible borate ester bond, and borax is used as a catalyst and dynamic crosslinking agent, expanding the design, formulation, and development possibilities and making it suitable for new hydrogel systems [56]. This prepared hydrogel is favorable for applications in wound dressings. More importantly, it provides a novel idea that is superior to the imine bond. The excellent self-healing ability of the borate ester bond has been demonstrated in the field of wound repair.

**Figure 6:**
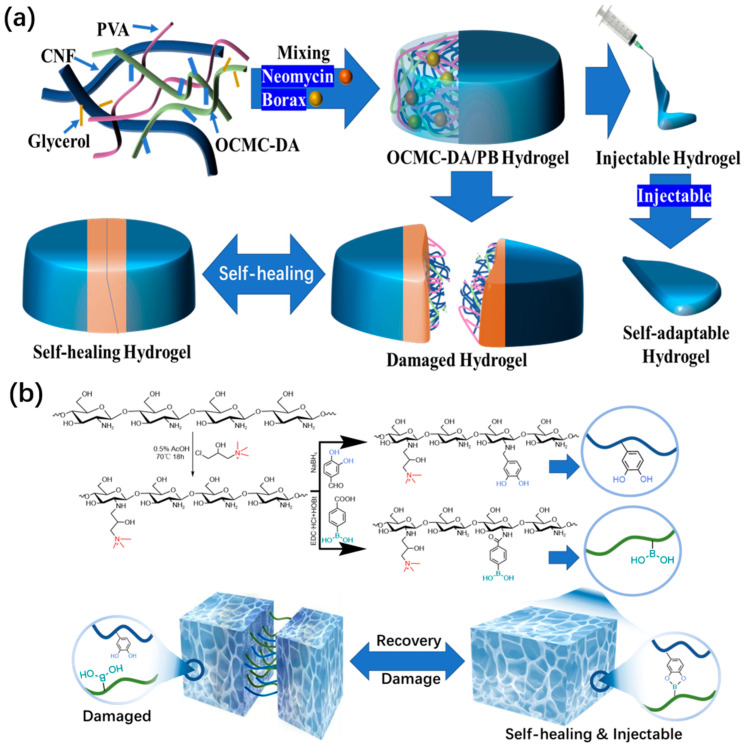
(**a**) Schematic illustration and microscopic structure of OCMC-DA/PB hydrogel and an application diagram of a self-healing and injectable hydrogel. (**b**) Schematic diagram of QCS-PC hydrogel preparation and the application of self-healing and injectable properties.

In order to demonstrate the advantages of borate bonds in the preparation of self-healing functional hydrogel dressing, Zhong et al. [57] used dopamine-grafted oxidized carboxymethyl cellulose (OCMC-DA) and cellulose nanofibers (CNF) to build a dynamic reversible borate ester bond to produce a hydrogel dressing that could quickly complete self-healing in 12 s and had certain injectability (Figure 6a). This type of excellent hydrogel dressing also accomplishes the function of degradation. The same research group has other achievements in the research of hydrogel dressings [58]. Through the dynamic covalent bond between boric acid and a catechol group, the quaternized chitosan can be loaded with epigallocatechin-3-gallate (EGCG), which can be used to meet the requirement of rapid self-healing (Figure 6b). Within 5 min, a hydrogel dressing that has been cut can be automatically reintegrated into one piece. If the injury is just a scratch, the scratch will vanish entirely from the healing interface after 20 s, realizing complete recovery. The adaptability and durability of this hydrogel as a wound dressing are significantly enhanced by its strong self-healing capacity. Compared to the aforementioned hydrogel dressings, its injectable properties are superior. Moreover, this hydrogel dressing also has antioxidant properties and a powerful bactericidal effect.

**Figure 7:**
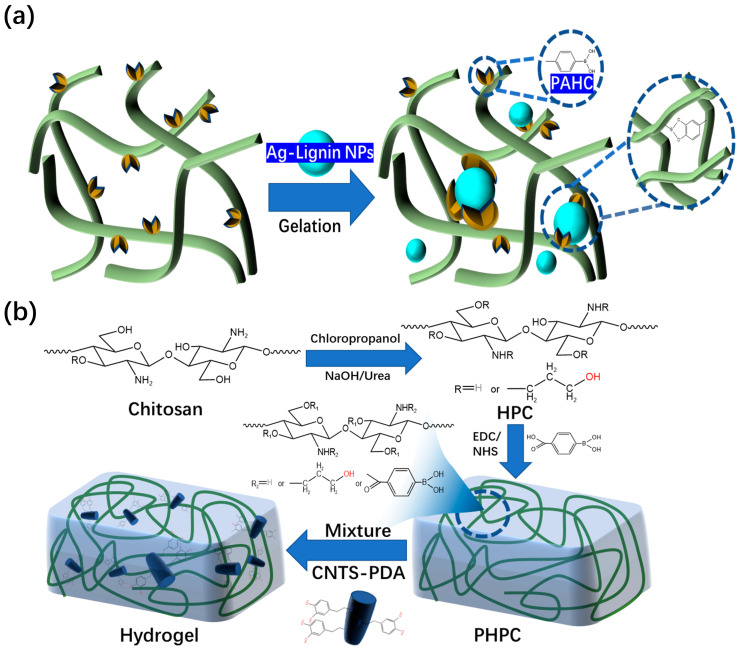
(**a**) Microstructure of hydrogels composed of PAHC and lignin-reduced Ag NPs. (**b**) Preparation mechanism of PHPC-CNT hydrogel dressing and its microscopic model.

Other research groups have studied this type of hydrogel dressings. Deng et al. [59] found that hydroxypropyl cellulose and phenylboronic acid-modified hydrogels could be constructed through dynamic borate bonds (Figure 7a). The prepared hydrogel dressing has excellent self-healing performance. The PAHC (phenylboric acid-modified hydroxypropyl cellulose) hydrogel completely healed after 10 min, which proves that it has excellent self-healing ability. Through continuous research, the research team has made preliminary achievements in the research and development of self-healing and injectable dynamic reversible gel dressings. However, the self-healing time of these dressings still needs to be improved. In 2022, Deng’s group [60] grafted 4-carboxylphenylboronic acid onto the molecular chain of hydroxypropyl chitosan (HPC) through amidation. The dynamic borate ester bonds are formed between phenylboronic acid and the catechol structure of polydopamine (PDA)-modified carbon nanotubes (Figure 7b). The final product has excellent self-healing ability compared to the other self-healing hydrogel dressing products mentioned above. The new hydrogel dressing developed by Deng’s group in 2022 can heal itself immediately. The new product developed by this team is more suitable for wound repair than other self-healing hydrogel dressings mentioned previously. This is because the self-healing time of the dressing has been significantly reduced compared to its previous iterations.

The most obvious conclusion to be drawn from Table 1 in this section is that dynamic reversible hydrogel dressings made of borate ester bonds generally have better self-healing and injectability than those made of imine bonds.

### 2.2. Reversible Noncovalent Bonds

Dynamic reversible hydrogel dressings can also be composed of reversible noncovalent bonds, such as metal coordination bonds and hydrogen bonds. These interactions are usually always in the dynamic state of fracture and reorganization, which can achieve rapid and repeated recovery [61].

#### 2.2.1. Metal Coordination Bond

Hydrogels with self-healing properties were prepared by forming cross-linking points based on ligand–metal coordination bonds on the polymer skeleton. By choosing different ligands and metal ions, a variety of hydrogels with different mechanical strengths can be made to satisfy the self-healing requirements of various biomedical applications [62].

**Figure 8:**
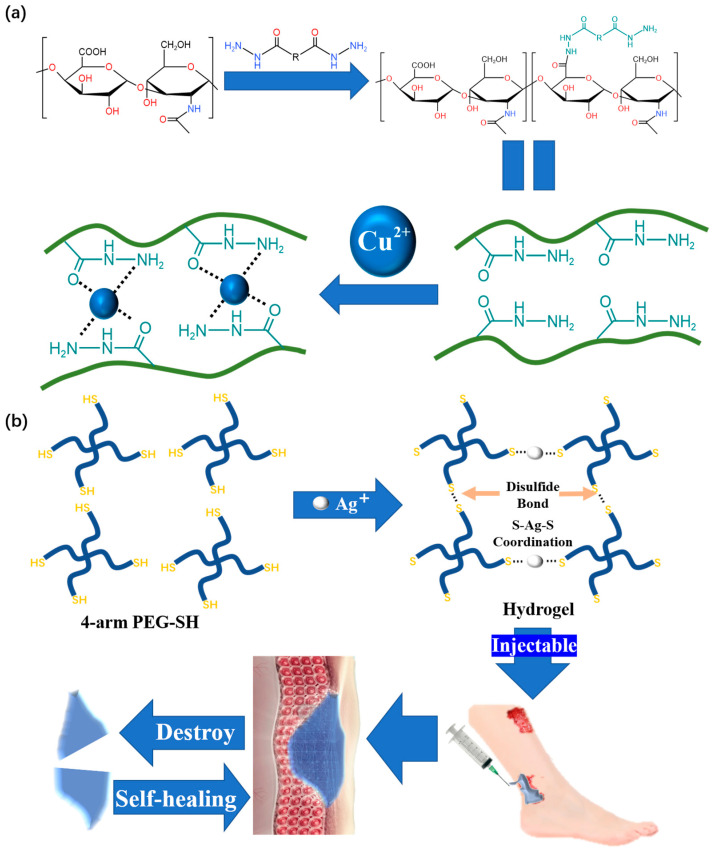
(**a**) The fabrication of HA hydrogels through hydrazide–metal coordination crosslinking and its microstructure. (**b**) Schematic illustration of the self-healing Ag(I)-thiol (Au–S) coordinative hydrogel and its self-healing and injectable applications.

Copper ions are a common material used for metal coordination crosslinking in reams of research. Using hydrazide group as ligand and Cu^2+^ as coordination center, Qian et al. [63] developed a copper–hydrazide-coordinated, multifunctional hyaluronan hydrogel, and the hyaluronic acid (HA)-Cu hydrogel shows practical versatility, including excellent self-healing and injectability (Figure 8a). Self-healing is achieved after 0.5 h, and the self-healing hydrogel can even be stretched during this time. From the perspective of hydrogel restoration, the HA-Cu hydrogel also has good injectability. Most importantly, this product is not restricted to only reacting in acidic conditions. This is the first report on hydrogels prepared by aliphatic hydrazine–metal coordination crosslinking for wound treatment.

We should also note that silver is frequently used for metal coordination crosslinking. Compared with Cu, Ag is more suitable for wound treatment due to its wider antibacterial spectrum and stronger antibacterial ability [64]. The hydrogel developed by Chen [65], which can be used for wound treatment, is prepared by the coordination crosslinking of dobby thiopenyl glycol (SH-PEG) and silver nitrate (AgNO_3_) (Figure 8b). Due to the dynamic nature of the Ag-S coordination bond, the obtained coordination hydrogels have good self-healing ability and injectability. At room temperature, the hydrogel shows excellent self-healing performance by restoring its original state from an incision within 15 min. A unique feature of the prepared hydrogel is its ability to have its mechanical strength adjusted, which is useful for adapting to various wound conditions.

**Figure 9:**
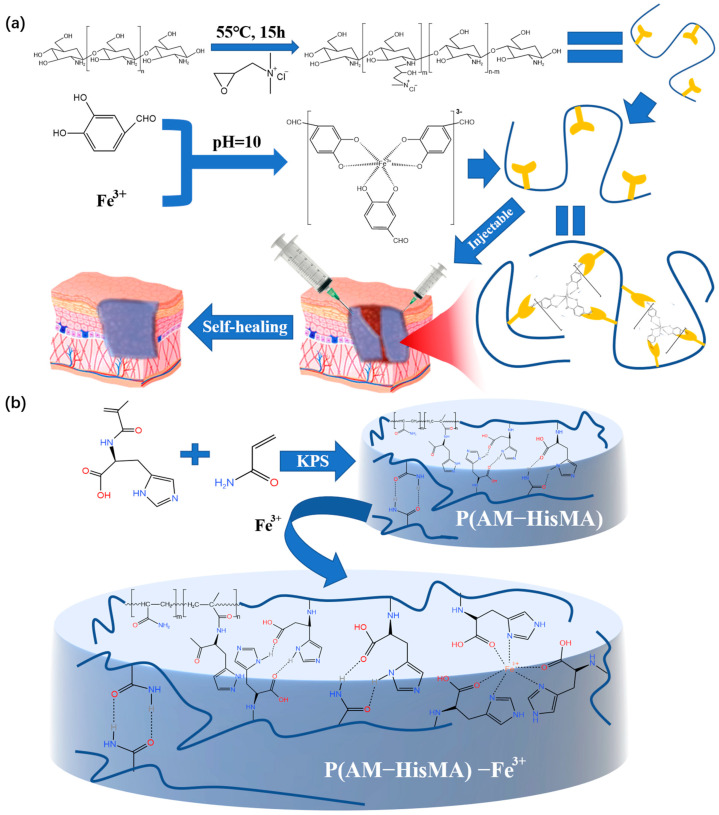
(**a**) The process of preparing double-dynamic-bond crosslinked hydrogels and the application of the product in medicine. (**b**) The preparation and the network structure of the P(AM-HisMA) −Fe^3+^ hydrogel dressing.

Fe^3+^ is very common, and the raw materials are cheap. In addition, Fe^3+^ can also be converted into Fe^2+^ in an acidic environment [66] (human skin is generally weakly acidic), which is helpful for the blood supply in the human body during wound healing. Moreover, this crosslinking method is environmentally friendly, simple, and universal. Based on the application of metal coordination bonds to prepare hydrogel dressings, the double dynamic bond crosslinked self-healing hydrogel prepared by Liang’s team [67] used the tricomplex coordination between the catechol groups from Fe^3+^ and protocatechualdehyde (PA) to produce hydrogels with excellent self-healing performance and injectability (Figure 9a). The resulting hydrogel dressing is intelligent and the wound repair efficiency is improved. The performance test revealed that the hydrogel could be fully repaired after 30 min of room temperature exposure and that its tensile capacity could be restored after an additional hour. It was proven that the reversible non-covalent cross-linked hydrogel dressing prepared by metal coordination has a certain self-healing ability, but the healing efficiency still needs to be improved.

Wound repair requires fast self-healing of the dressing in order to better repair the wound. Therefore, after using histidine methacrylamide (HisMA) and acrylamide (AM) to form hydrogel, Zhang [68] added Fe^3+^ ions into a P(AM-HisMA) hydrogel to prepare a P(AM-HisMA)-Fe^3+^ hydrogel dressing with good injectability and self-healing ability (Figure 9b). It is worth noting that the self-healing speed is greatly improved compared to the hydrogel without Fe^3+^. P(AM-HisMA)-Fe^3+^ hydrogel completely heals in 5 min; however, the P(AM-HisMA) hydrogels without Fe^3+^ crosslinking take 24 h to achieve self-healing. Since its capacity for self-healing is an important reference scheme for this kind of hydrogel dressing, it merits special attention.

#### 2.2.2. Hydrogen Bond

Another type of dynamic noncovalent bond, the hydrogen bond [69], is a noncovalent bond that can be destroyed at high temperature and reformed at low temperature. Therefore, hydrogen bonds can be used to prepare self-healing hydrogel dressings.

**Figure 10:**
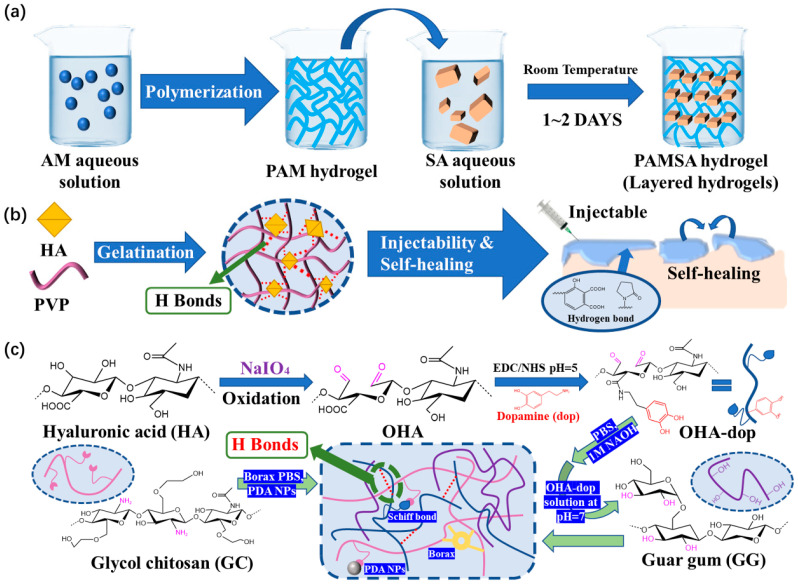
(**a**) Schematic diagram of PAMSA hydrogel preparation based on PAM and SA. (**b**) Schematic diagram of HPC hydrogel crosslinked by HA in an ultrafast process and the possible hydrogen bonding structure of HPC hydrogels. (**c**) The preparation and microscopic schematic diagrams of the OHAdop/GG + GC/borax hydrogel and the OHAdop/GG + GC/borax/PDA hydrogel.

In order to improve the self-healing ability and injectability of functional hydrogel dressings, Zhao [70] developed a simple and fast method to prepare a new hydrogel. This hydrogel is composed of sodium alginate (SA) and polyacrylamide (PAM). Systematic characterization revealed the formation mechanism of a layered structure through hydrogen bonding (Figure 10a). In addition, it takes 0.5 h to achieve self-healing. Hydrogen bonds allow for the recovery of the mechanical properties of hydrogels with or without the presence of water.

There are also better research results on the self-healing ability and injectability of hydrogel dressings. Among dynamic crosslinking hydrogel dressings with these properties, the humic acid/polyvinylpyrrolidone (PVP) complex hydrogel dressings prepared by Yu et al. [71] are driven by the hydrogen bonds between humic acid and PVP, and these bonds are easy to form and adjust (Figure 10b). The dynamic reversible hydrogen bonds distributed in the hydrogel dressing promoted the formation of a dynamic cross-linking network. The viscosity of the humic acid/PVP complex hydrogel significantly decreases as the shear rate rises, improving its injectability. At the same time, this hydrogel is endowed with self-healing performance that can achieve complete self-repair in 10 min.

Because the force of hydrogen bonds is generally weaker than some of the dynamic reversible bonds discussed above, hydrogen bonds often do not appear alone; rather, hydrogels are constructed combing hydrogen bonds with other dynamic cross-linking bonds. Guo et al. [72] designed and prepared a polysaccharide-based adhesive hydrogel, in which dynamic hydrogen bonding between catechol-modified oxidized hyaluronic acid (OHAdop) or hydroxide groups in guar gum led to reversible crosslinking (Figure 10c). As soon as the two parts come into contact, hydrogen interaction occurs, causing the fragments to self-heal and unite to form a single hydrogel that can be easily combined with other parts. This hydrogel dressing heals itself extremely quickly. It has high application value and competitiveness in the market.

## 3. Modified Hydrogel Dressings

Nowadays, it is popular to make new hydrogel dressings using modified methods because existing hydrogel dressings are unable to meet the needs of combined treatment, including continuous drug release according to the physiological conditions of the wound [73]. Modification is often carried out through physical or chemical pathways to change the form or properties of the materials [74]. Hydrogels prepared by modified methods, such as through changing the composition of the object or the preparation process, gives the hydrogel excellent performance in many aspects [75]. These attempts provide support for the research and development of more hydrogel dressings with more functions and better efficacy through the synthesis and testing of new hydrogel dressings that combine the advantages of various substances so that more hydrogels can enter the clinic and be widely used.

**Figure 11:**
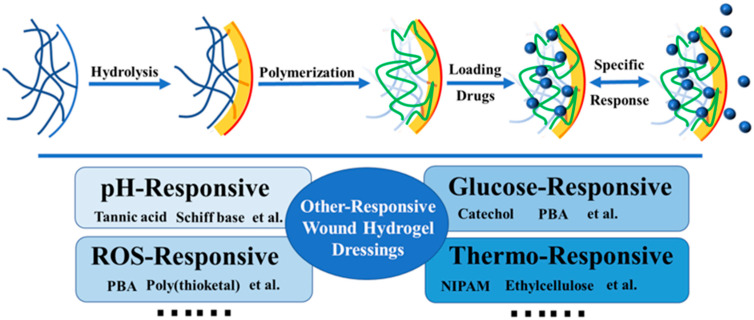
The general method of drug release of stimuli-responsive modified hydrogel dressings.

The appearance of the wound while healing is a dynamic process, accompanied by different types of chemical and physical changes. As in Table 2, pH response, reactive oxygen species (ROS) response, thermal response, glucose response, and many other factors will affect wound healing. Because they can actively alter the wound environment to maintain an appropriate healing process, wound dressings that can react to these changes are considered targeted and intelligent [76]. Therefore, modified functional hydrogel dressings can be designed to have many special properties, especially to realize the controllable transportation of drugs through the hydrogel dressing [77]. Based on the above-mentioned response signals, we will focus on the common modified stimuli-responsive hydrogel dressings developed in recent years (Figure 11).

### 3.1. pH-Responsive Hydrogel Dressings

**Figure 12:**
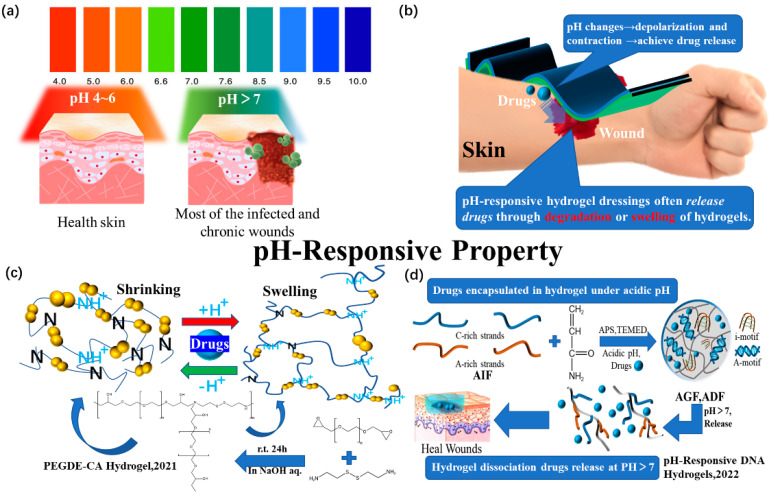
(**a**) pH differences between healthy skin and infected or chronic wounds. (**b**) Application diagram of pH-responsive hydrogel dressings. (**c**) Schematic diagram of Komatsu’s team’s (2021) study on the mechanism of the hydrogel dressing. (**d**) Schematic diagram of Hu’s team’s (2022) study on the mechanism of the hydrogel dressing.

Healthy and normal skin [89] is acidic with a pH between 4 and 6 (Figure 12a). After an injury, the pH value of the wound increases due to microvascular leakage and underlying tissue exposure, with pH values generally greater than 7. This alkaline environment is conducive to bacterial infection [90]. pH-responsive hydrogels are widely studied as drug transport carriers because these hydrogel dressings can respond to changes in the body’s pH environment. This stimulation can change the drug loading and release behavior through mesh size control and hydrophobic/electrostatic interactions (Figure 12b) of the drug with the hydrogel’s polymer network.

A pH-sensitive hydrogel was prepared using the ring opening reaction of the epoxide group in poly(ethylene glycol) diglycidyl ether (PEGDE) with the amino group of cystamine (CA) as the crosslinking agent [78]. The hydrogel prepared by modification enters a swelled and distended state due to amino protonation under acidic conditions, allowing for the slow diffusion of amino acid drugs from the hydrogel (Figure 12c). These results indicate that the drug proteins in the hydrogel can be completely released under reducing conditions because wound healing provides ideal release characteristics.

Hu et al. [79] formed a copolymer with an acrylamide monomer through the free radical polymerization of oligonucleotides rich in adenine (A) and cytosine (C). In an acidic environment (pH 1.2~6.0), the resulting copolymer forms a hydrogel (Figure 12d). The hydrogel will disintegrate and release drugs at a pH of 7.2, which is appropriate for the pH value of many types of wounds. This hydrogel can also be used as a dressing to release drugs in a targeted manner.

Because the pH value is the response signal, the drug released from the modified hydrogel dressing can accurately act on the wound without affecting the normal skin [91]. In this regard, our research group has also carried out similar research [92,93], and there are also other relevant research papers to support this view.

### 3.2. ROS-Responsive Hydrogel Dressings

**Figure 13:**
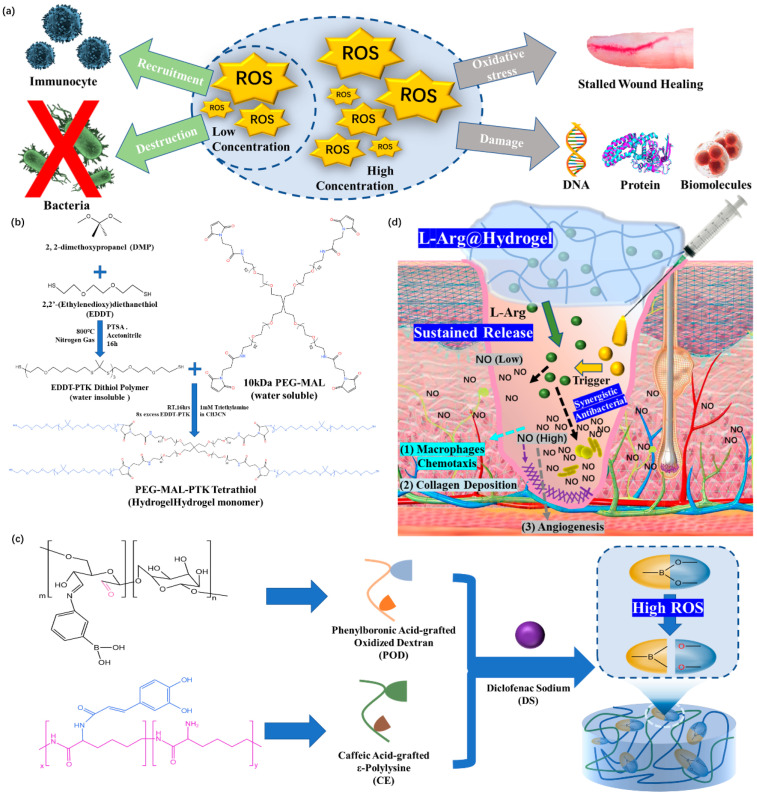
(**a**) Schematic diagram of the effect of ROS concentration on wound repair. (**b**) Synthesis scheme of PEG-MAL-PTK in hydrogel dressing. (**c**) Schematic diagram of the DS@MF embedded POD/CE hydrogels and its working mechanism. (**d**) Schematic diagram of wound treatment using L-Arg@Hydrogel.

ROS are produced by wounds or bacterial infections in the wound environment [94]. The accumulation of ROS in the wound will lead to serious inflammatory reactions, reduce the regenerative ability of endogenous stem cells and macrophages, and inhibit wound repair [95]. Therefore, it is important to determine the content of ROS in the wound environment as an indicator of controlled drug release (Figure 13a). To design a hydrogel dressing that can be reactive to ROS and maintain controllable drug release is a good strategy for clinical wound repair. Utilizing the differences in the biochemical environment between the wounded skin and the healthy skin, this selective release of the drugs is made possible.

On one hand, ROS content is an important reaction signal for drug release. When used as a wound dressing, ROS-responsive hydrogel can release a specific drug while also self-degrading on its own. There is no need to remove the dressing afterwards, thus avoiding secondary injury. Martin [80] successfully synthesized poly(ethylene glycol) (PEG)-based hydrogels crosslinked with ROS-degradable poly(thioketal) (PTK) polymers through thiol-maleimide (Figure 13b). In addition to using ROS as a response signal, this hydrogel can also self-degrade, which leads to the realization of drug release. Within the first 24 h, the majority of the drugs in the product were released.

Too rapid a drug release prevents the dressing from fully fulfilling the goal of speeding wound healing [96]. Phenylboronic acid-grafted oxidized dextran and caffeic acid-grafted ε-polylysine were used to successfully prepare modified hydrogels with ROS responsiveness (Figure 13c). It can also be used as a wound dressing in drug transportation. The modified grafted component was hydrolyzed, and the hydrogel network was destroyed under the oxidation state. Once the network collapses, the drug will be exposed to the wound itself. The wound area of the group covered with the ROS-sensitive hydrogel dressing showed a faster contraction rate than that of the blank group, and the wound treated with this hydrogel dressing was also basically healed after 14 days [81]. Most importantly, the hydrogel dressing can be released continuously and slowly within 7 days, improving the therapeutic ability while reducing the frequency of drug replacement.

On the other hand, drugs with controlled release could also minimize harm to the human body. It is very important that functional hydrogel dressings have the ability of drug release with high sensitivity. This can effectively avoid adverse effects on the human body caused by factors inducing drug release. It has been found that nitric oxide (NO) plays a key role in regulating various wound healing processes, which is beneficial to cell proliferation, the antibacterial effect, and angiogenesis [97]. In a study on NO’s role in wound healing, Yu [82] reported that an L-Arginine@hydrogel dressing could use different ROS concentrations to control the speed of NO release. This feature enabled the hydrogel to expedite the healing process of open wounds without causing any irritation to the patient. By reacting with L-Arg, a low level of H_2_O_2_ is introduced to trigger the release of NO in the dressing, thus realizing a drug release rate that is subject to subjective human control (Figure 13d). This is a step closer to a more scientific wound repair effect.

### 3.3. Other Types of Responsive Hydrogel Dressings

The increasing number of clinical cases involving trauma repair has led to a rise in the number of different kinds of trauma, such as sports injuries, chronic wounds related to diabetes, burns, and more. Modified functional hydrogel dressings with specific drug delivery systems for different trauma have very important research significance [98]. Scholars have developed new types of modified hydrogel dressings, which could be responsive to glucose, mechanical properties and heat.

#### 3.3.1. Glucose-Responsive Hydrogel Dressings

**Figure 14:**
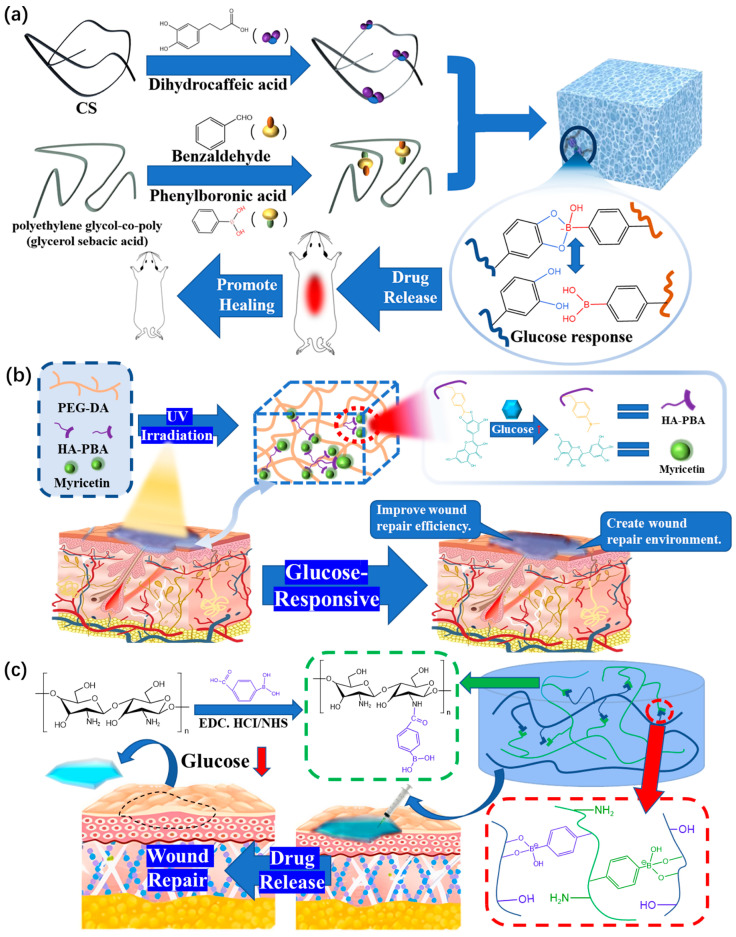
(**a**) Schematic diagram of the preparation and drug release of PC/GO/Met hydrogel. (**b**) Diagram illustrating the PHM hydrogel platform for wound healing and the microstructure of hydrogel dressings. (**c**) Schematic diagram of the preparation mechanism and the mechanism of CBP/GMs@Cel&INS hydrogel dressing for drug release.

In the treatment of chronic wounds in diabetes, Liang et al. [99] successfully prepared a new hydrogel dressing using glucose as the response signal through the combination of catechol and phenylboric acid (Figure 14a). The principle here is that glucose can compete for the combination between catechol and phenylboric acid, which makes the network part of the hydrogel dissociate and release the corresponding drug. This provides a new preparation method for the research and development of functional hydrogel dressings. The final cumulative drug release of the dressing was 23.3% higher than that of the glucose-free group.

At the same time, according to other needs of wound treatment for diabetes, some scholars have also used glucose as the corresponding signal to create other repair mechanisms. Xu’s team [100] modified phenylboronic acid (PBA), which has unique glucose sensitivity, onto a hyaluronic acid (HA) chain base to prepare a new hybrid hydrogel (PEG-DA/HA-PBA) dressing (Figure 14b). This modification realizes the release of myricetin (MY) in the treatment of diabetic wounds. In the wound healing ability test, the recovery rate of the control group was 65.04% on the 20th day, while the recovery rate of the PEG-DA/MY group increased to 83.62%, showing an improvement in the wound healing ability.

Zhou [85] used a modified glucose-responsive hydrogel dressing prepared by polyvinyl alcohol (PVA) and chitosan-grafted phenylboric acid (CS-BA). Its network regulates drug release (as shown in Figure 14c) through the glucose-induced cleavage of phenylborate ester bonds, leading to the hydrolysis and release of drugs. Importantly, the hydrogel dressing enables the real-time controlled release of drugs based on the conditions of the wound, thus improving the therapeutic efficiency of the drugs.

#### 3.3.2. Pressure-Responsive Hydrogel Dressings

**Figure 15:**
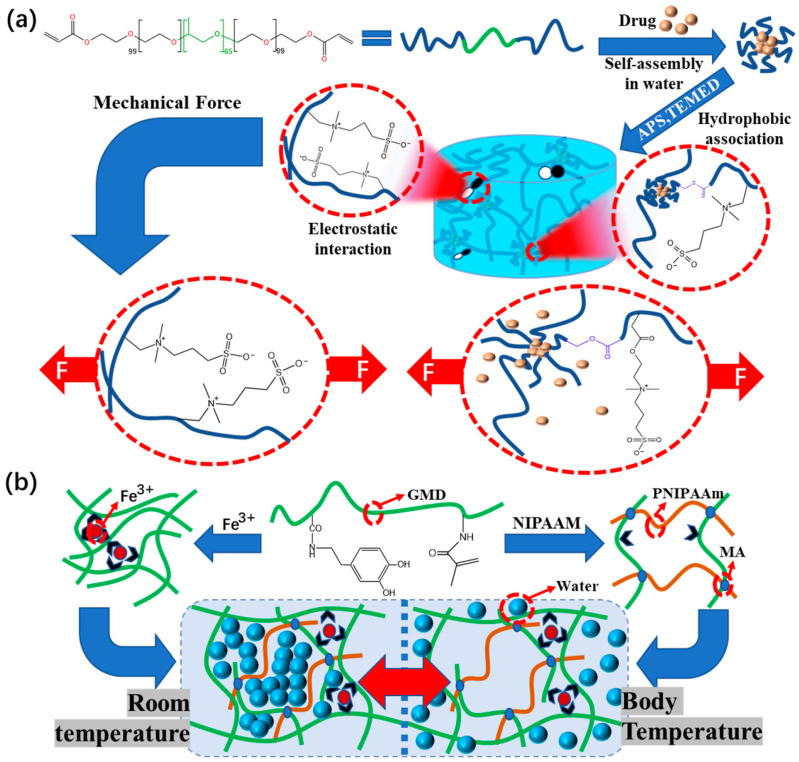
(**a**) Schematic diagram of hydrogel network deformation and drug release caused by pressure changes caused by mechanical force. (**b**) Schematic diagram of the structure and thermo-sensitive mechanism of the hydrogel dressing.

In the treatment of joint wounds, the skin of the fingers and elbows often deforms during various daily behaviors. For this very common wound type, researchers take this deformation as the response signal for drug release. A pressure-responsive hydrogel dressing was designed based on the excellent antibacterial and conductive properties of imidazole-based ionic liquids [87]. This hydrogel dressing can be used with good sensitivity to pressure changes. Fang [88] prepared a pressure-responsive poly(sulfobetaine methacrylate) hydrogel dressing (Figure 15a). Under tensile and compressive forces, the hydrogel network of the modified hydrogel dressing is deformed due to external mechanical forces, resulting in the release of drug molecules into the hydrogel matrix. Finally, it diffuses into the external aqueous environment, and the drug release rate can also be controlled.

#### 3.3.3. Thermo-Responsive Hydrogel Dressings

Inflammation in a wound can cause symptoms such as redness, swelling, fever, and pain. These symptoms stimulate the expansion of blood vessels in local tissues, accelerate blood flow, enhance local catabolism, generate heat, and slightly increase the temperature near the wound. In view of this characteristic of the wound, Hou et al. [101] designed a graft-modified thermosensitive hydrogel dressing formed by catechol Fe^3+^ and NIPAAm (N-isopropylacrylamide), which has an intelligent drug transport system (Figure 15b). The lower critical solution temperature (LCST) of the PNIPAAm thermosensitive hydrogel in the dressing is close to the physiological temperature of the human body, and it can respond quickly to external stimuli to achieve controlled drug release.

In the current market, among the hydrogel products that have emerged with related functions, a large number of clinical manifestations have also proven the importance of such hydrogel dressings. For instance, *Solaraze^®^* [102] has successfully prepared excellent drug carriers by encapsulating drugs with HA. In clinical trials, 2 g *Solaraze^®^* was administered three times a day, applied locally to the legs of healthy subjects for a total of six days. Diclofenac was detected in plasma, with average bioavailability parameters of AUC_0−t_ 9 ± 19 ng/mL (mean ± SD), C_max_ 4 ± 5 ng/mL, and T_max_ 4.5 ± 8 h. This indicates the sustained and effective drug release ability of the products.

Another example of a commercial application of functional hydrogel dressing is a dressing controlling the delivery of a drug named *Prontosan^®^* [103]. In clinical practice, seven out of ten patients with similar injuries who participated in the test achieved efficient wound repair within three weeks, demonstrating the effectiveness of the functional hydrogel dressing in transporting drugs and achieving wound repair in a short time.

These cases provide strong support for the feasibility and practical application of the design concept of functional hydrogel dressings in clinical settings.

## 4. Composite Hydrogel Dressings

**Figure 16:**
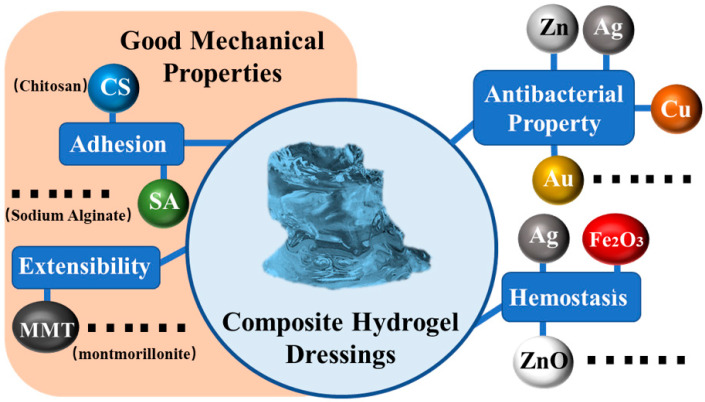
The effect of chemical particles on wound repair in composite hydrogel dressings.

In recent years, nanocomposite hydrogels with a polymer matrix doped with certain particles have received extensive attention [104]. Different chemical particles have different properties and can be used in the process of wound repair. In particular, composite hydrogel dressings have strong antibacterial [105], hemostatic [106], and mechanical [107] qualities that are crucial in the study of functional hydrogel dressings. To maximize their functions, functional hydrogel dressings made from composite hydrogels were loaded with various particles using various methods (Figure 16). Early functional hydrogel dressings frequently used this approach to add new capabilities to a product. As shown in Figure 13, in order for a composite hydrogel dressing to achieve functional development, different types of nanoparticles were embedded into the bulk hydrogel framework [108], and a variety of composite hydrogel dressing materials with a uniform particle distribution were developed. This was shown to greatly expand the functionality of functional hydrogel dressings.

Simultaneously, according to their most representative functions, composite hydrogel dressing can be divided into three categories: antibacterial composite hydrogel dressings, hemostatic composite hydrogel dressings, and compound hydrogel dressings with the function of improving mechanical properties. These three categories will serve as a starting point to describe the development of the functional research on composite hydrogel dressings in more detail.

### 4.1. Antibacterial Composite Hydrogel Dressings

**Figure 17:**
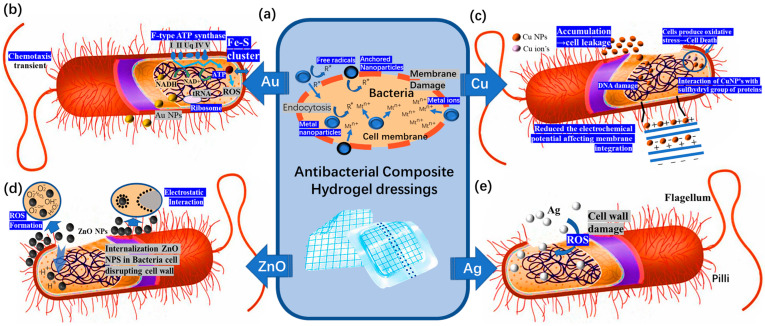
The mechanism of the antibacterial effect of different kinds of metal elements. (**a**) Antibacterial mechanism of composite hydrogel dressing using chemical particles. (**b**) The antibacterial principle of Au. (**c**) The antibacterial principle of Cu. (**d**) The antibacterial principle of ZnO. (**e**) The antibacterial principle of Ag.

Bacterial infection is one of the most common problems in wound healing [109]. When a wound is infected, the bacteria will continue to slow down the inflammatory stage’s healing process and may potentially cause complications [110]. Therefore, hydrogel dressings have good antibacterial properties and thus have important clinical significance [111]. Although there are some common hydrogel copolymers that can provide some antibacterial properties (Figure 17a,d), researchers should also develop advanced functional hydrogel dressings with more specificity and greater wound healing efficiency by preparing composite hydrogel dressings according to the characteristics of microbial infections.

It is well known that metals and their compounds are essential trace elements for the human body [112], including copper, zinc, manganese, chromium, etc. These metal elements participate in some physiological processes in the body and maintain the normal functions of the human body. Meanwhile, hydrogels [113] can be compatible with human systems without risk of toxicity or immunogenicity. Therefore, the combination of metals and hydrogels will make the new product an ideal candidate for wound healing.

Metals or metal compounds can effectively enhance the antibacterial activity of composite hydrogel dressings. The research works listed in Table 3 combined typical metals or metal compounds with hydrogel dressing with the aim to enhance the dressing’s antibacterial activity.

**Figure 18:**
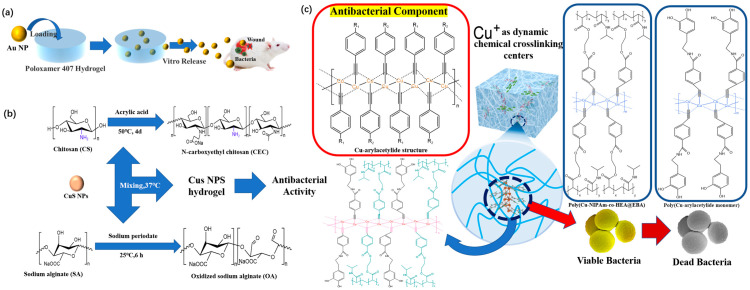
(**a**) The strategy and in vitro release model of Au loaded into poloxamer 407 hydrogel. (**b**) Preparation scheme and schematic diagram of CuS NPs hydrogel. (**c**) Preparation mechanism and antibacterial mechanism of poly(Cu-arylacetylide)-derived hydrogel.

In the early research, most antibiotics were modified on the surface of gold nanoparticles, and the gold nanoparticles (Figure 17b) made the antibiotics show superior antibacterial activity than those without modification [120]. To improve the antibacterial ability, Mahmoud et al. [116] added prepared AuNPs as antibacterial substances to hydrogels to prepare hydrogel dressings. At appropriate concentrations, AuNPs accelerate wound healing by inhibiting the growth of multidrug-resistant bacteria (Figure 18a). It has been proven that AuNPs can not only be used as drug conjugates but can also be used directly and independently to improve the antibacterial ability of a product. However, the cost of gold is too high to be put into the market in the near future, and more particles with lower costs and excellent antibacterial effects are needed to prepare composite hydrogel dressings. Therefore, other metals or metal compounds have gradually gained the attention of researchers. Xie et al. [121] also found that reducing the size of the gold nanoparticles is conducive to obtaining better antibacterial properties. This is also applicable to the preparation of gold and other nanoparticles for antibacterial composite hydrogel dressings.

Considering that gold has lower availability compared to copper, the long-term production costs of the gold smelting process is significantly higher. In contrast, the copper smelting process is well-established and mature, resulting in lower production costs compared to gold [122]. This perspective highlights that copper is a more cost-effective option for the large-scale production and commercialization of hydrogel dressings compared to gold. Therefore, copper is more appropriate than gold for use as an antibacterial component in composite hydrogel dressings (Figure 17c). The oxidation process in cells is catalyzed by copper ions, which can inhibit and eliminate viruses and bacteria. Kong [123] et al. also prepared a composite hydrogel with good antibacterial properties by using mixed antibacterial particles. This hydrogel, composed of modified CuS NPs, was synthesized as a multifunctional dressing to improve the dressing’s antibacterial efficiency (Figure 18b). Its antibacterial efficiency against *Staphylococcus aureus* and *Escherichia coli* can reach to 99.62% and 99.67%, respectively. This can effectively destroy the bacterial cell wall in the clinical treatment process and ultimately lead to changes in bacterial morphology and finally death. This dressing therefore meets the requirements for the antibacterial performance of wound dressings. However, hydrogel dressings made of copper compounds need a certain amount of light to achieve this ideal antibacterial effect.

Xia et al. [124] reported for the first time that poly(Cu-arylacetylide)-derived hydrogels, known as poly(Cu-N-isopropylacrylamide) (poly(Cu-NIPAm)) hydrogels, are different from traditional hydrogel materials due to their special copper arylacetylate chains. These hydrogels can have excellent antibacterial properties without additional conditions needing to be met (Figure 18c). In the antibacterial tests, the hydrogel produced minute amounts of copper ions from the a-chain, which disrupted the biochemical cell pathway of the bacteria and led to their demise. In HCuM_x−y_ (formed by the Cu^1+^, M_1_, and arylacetylide groups on pNIPAm side chains, where x is the molar ratio of the Cu to NIPAm and y is the molar ratio of the M_1_ to the total arylacetylide groups of M_1_ and pNIPAm side chains), the bacterial treatment solution is challenging to observe. The concentration of these bacteria in the pNIPAm hydrogel treatment fluid is seven times greater than that in standard hydrogel under the same circumstances. This high-quality antibacterial property has broad prospects for the application of wound dressings.

However, although Cu has a reduced cost, there is still more room for improvement in antibacterial performance [125]. Copper can also damage red blood cells and cause hemolysis and anemia.

Compared to the above-mentioned gold and copper metals and their compounds, silver is a better choice for composite hydrogel dressings. Among the typical metals, the application of Ag (Figure 17e) in antibacterial wound research has a long history. Ag is cheaper than gold [126], and Ag has better germicidal efficacy than Cu at the same concentration [127]. The chemical properties of Ag are very stable, and its corrosion resistance is also strong. Ag can improve the antibacterial activity against *Staphylococcus aureus*, *Escherichia coli*, and other bacteria and reduce the infection rate of the wound.

**Figure 19:**
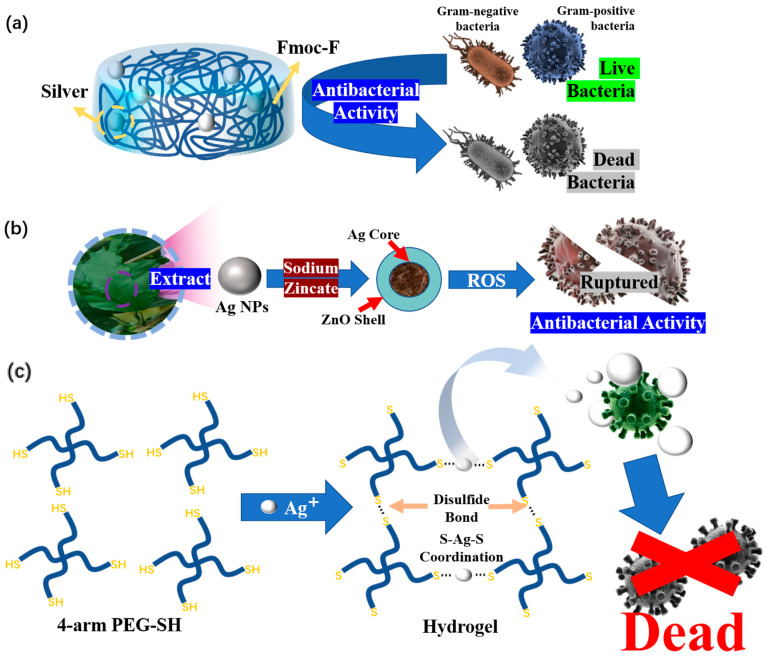
(**a**) Composition and antibacterial mechanism of Fmoc-F-based, self-assembled, supramolecular hydrogel dressing. (**b**) Schematic illustration of the Ag(I)-thiol (Au–S) hydrogel and its antibacterial mechanism. (**c**) Preparation mechanism and antibacterial mechanism of PVP/PVA/Ag@ZnO hydrogel dressings.

Many research advances have been made regarding composite hydrogel dressings prepared using heavy metals such as Au, Ag and Cu. However, due to the shortcomings of these heavy metals, their long-term use as wound dressings is greatly affected. Scholars have found that composite hydrogel dressings can be prepared by combining certain antibacterial particles with coordination methods [128]. Composite hydrogel dressing prepared in this wat are more stable than composite hydrogel dressings prepared by direct injection and can also make the antibacterial effect of the particles used more sustainable. Zhao et al. [129] prepared a self-assembled supramolecular hydrogel based on Fluorenylmethyloxycarbonyl-phenylalanine (Fmoc-F) through the simple incorporation of silver ions, which contributes to the antibacterial activity (Figure 19a). This is one of the simplest ways to prepare composite hydrogel dressings containing silver. Chen et al. [65] synthesized hydrogels through the coordination and cross-linking of thiopolyethylene glycol (SH-PEG) and AgNO_3_ and loaded them with desferrioxamine (DFO) (Figure 19b). This hydrogel dressing can exert antibacterial and infection prevention effects because of the reversible Ag-S bond produced by cross-linking SH-PEG and AgNO_3_ and the interaction between the hydrogel and sulfur-containing proteins in the bacterial cell membrane. It is noteworthy that this hydrogel dressing loaded with DFO will neither induce cytotoxicity nor adversely affect the wound.

Because of the potential adverse effect of heavy metals and the super permeability of nanoparticles [130], the widespread use of silver nanoparticles will also have adverse effects on human beings. Now, more research is focusing on combining other metals and other compounds to prepare composite hydrogel dressings, so that the shortcomings of both materials can be improved. For example, the toxicity of ZnO NPs [131] (Figure 17d) to healthy cells and tissues has always been the biggest obstacle to the development of ZnO-based therapies. Considering these problems, in Khan’s team [118], hibiscus sabdariffa was used in the processing of Ag NPs, followed by a coating of a thin layer of ZnO on biogenic Ag NPs to minimize their toxicity towards mammalian cells (Figure 19c). This provides a better approach for the future development of antibacterial composite hydrogel dressings. We also have reason to believe that this will be an important research direction in the future.

In the current market, *Suprasorb^®^ A + Ag* [132] is a hydrogel dressing that employs the design idea of loading the hydrogel with antibacterial chemical particles to achieve a high antibacterial efficiency. After 24 h, such a dressing was able to harnesses the excellent antibacterial ability of silver (Ag). In clinical trials involving 99 individuals with varying degrees of infection, in vitro observations showed antibacterial effects related to ionic silver from alginate silver fibers, which remained stable for up to 7 days.

### 4.2. Hemostatic Composite Hydrogel Dressings

Hemostasis is an important part of wound repair. Uncontrolled bleeding during the healing process is one of the most dangerous and potentially lethal issues in wound healing, which can delay healing and even result in death, especially in very critical circumstances [133]. It is very important to stop bleeding quickly. Coagulation time is the most intuitive way to evaluate the hemostatic performance of a hydrogel dressing.

**Figure 20:**
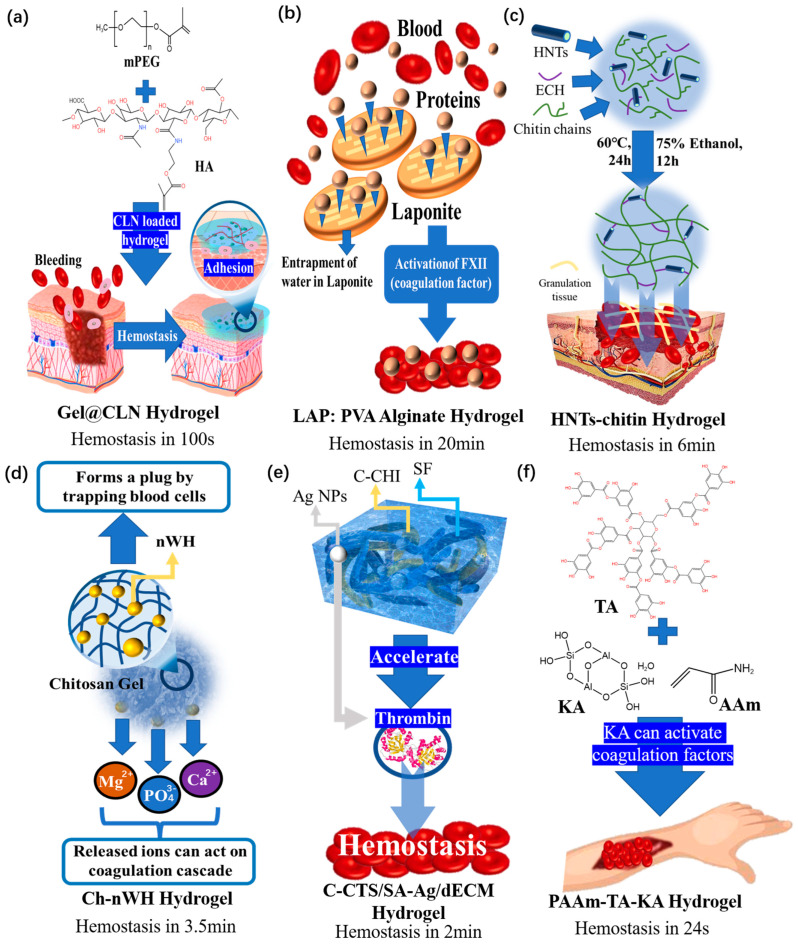
(**a**) Schematic diagram of the cross-linking process, hemostasis mechanism, and coagulation time of Gel@CLN hydrogel. (**b**) Schematic diagram of the cross-linking process, hemostasis mechanism, and coagulation time of LAP: PVA alginate hydrogel. (**c**) Schematic diagram of the cross-linking process, hemostasis mechanism, and coagulation time of HNTs-chitin hydrogel. (**d**) Schematic diagram of the cross-linking process, hemostasis mechanism, and coagulation time of Ch-nWH hydrogel. (**e**) Schematic diagram of the cross-linking process, hemostasis mechanism, and coagulation time of C-CTS/SA-Ag/dECM hydrogel. (**f**) Schematic diagram of the cross-linking process, hemostasis mechanism, and coagulation time of PAAm-TA-KA hydrogel.

On the one hand, many studies have demonstrated that loading non-metallic particles into hydrogels to increase cell adhesion can also result in improved hemostasis. For example, Zhu [134] used a hydrogel (Figure 20a) combined with methacrylated methoxy polyethylene glycol (mPEG-MA) and chlorhexidine diacetate (CHX)-loaded nanoparticles (CLNs) to stop bleeding in 100 s.

On the other hand, accelerating the clotting reaction is a better and more common method. In order to accelerate the blood coagulation reaction during wound repair, mineral particles that are helpful for hemostasis can be loaded to promote hemostasis. Golafshan [135] et al. successfully prepared a nanohybrid composite hydrogel of laponite: polyvinyl alcohol (PVA)-alginate (LAP:PVA Alginate). The hemostatic effect was improved by adding laponite, and it was found that the coagulation time was reduced to 20 min by increasing the content of alginate. The schematic diagram of the coagulation effect of Golafshan’s hydrogel dressing products is shown in Figure 20b. In comparison to the hydrogel dressings without additional particles, this new hydrogel causes the activation of coagulation factors, which reduces the time needed for coagulation.

Zhao et al. [136] used chitin as the matrix to construct a hydrogel (Figure 20c) that can stop bleeding within 6 min, and the main hemostatic ingredients, halloysite nanotubes (HNTs) and chitin, rarely cause immune rejection. Metal particles are often used to achieve hemostatic performance, and how to improve the hemostatic efficiency is also being studied constantly. To date, the hemostatic effect has entered the range of a few minutes. Pillai [137] Jack loaded 2% Ch–4% nWH (nano-whitlockite) to prepare a composite hydrogel (Figure 20d). This hydrogel was shown to have a good hemostatic effect, achieving coagulation in 3.5 ± 1.2 min by releasing Ca^2+^, Mg^2+^, and PO_4_^3−^ from nano-whitlockite.

Based on mussels and barnacles, Pan [138] introduced Ag NPs to develop a new composite hydrogel dressing (Figure 20e). In this hydrogel, Ag NPs can accelerate the production of thrombin by activating platelets and increasing the exposure of phosphatidylserine, thus improving the hemostatic ability. When the blood is in full contact with the hydrogel for 120 s, the liquid blood is converted into coagulated blood. This product makes composite hydrogel a promising emergency hemostatic dressing material.

There are also other particles that can help stop bleeding and have similar hemostatic efficiency to metal particles. For example, Fan [139] loaded kaolin into a PAAm (polyacrylamide)–tannic acid (TA)–kaolin (KA) hydrogel (Figure 20f), which greatly enhanced the coagulation reaction, thus reducing the coagulation time to 24–31 s. Because KA is the contact activator of the coagulation effect, the hemostasis speed is very fast, and this hydrogel has great potential in achieving hemostasis in arterial ruptures.

One feasible example of this preparation method is in clinical trials of existing products, specifically the *Gelatin LAPONITE nanocomposite hydrogel* [140]. In these trials, different concentrations of silicate were incorporated, and it was observed that products with silicate concentrations of 25%, 50%, and 75% reduced the coagulation time by 32%, 54%, and 77%, respectively. This demonstrates potential for commercial production and provides support for the viability of this approach.

### 4.3. Composite Hydrogel Dressings with Good Mechanical Properties

**Figure 21:**
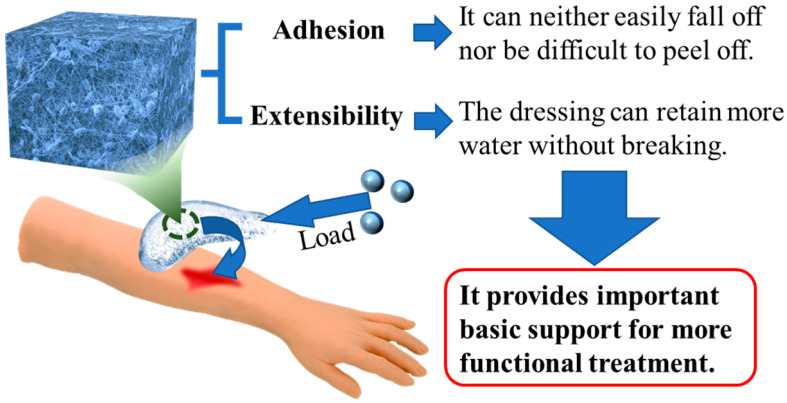
The effect of good mechanical properties on functional composite hydrogel dressings.

In the process of preparing traditional hydrogel dressings, researchers often find that traditional hydrogels have problems such as uneven network structures and a lack of energy dissipation mechanisms [141]. As a result, the mechanical properties of traditional hydrogels are poor and their adhesion to skin is weak, which limits their application in practical production and life to a large extent [142]. The tensile and adhesive capabilities of hydrogel can be enhanced in composite hydrogel dressings. Adding these new functions is a good way to improve the mechanical properties of hydrogels (Figure 21). By adding various nanomaterials to the hydrogel matrix as fillers, the adhesion and tensile mechanical properties of the nanocomposite hydrogel formed can be significantly improved. Thus, composite hydrogel dressings can also effectively improve the mechanical properties of standard dressings [143].

#### 4.3.1. Adhesion

In terms of adhesion, general hydrogel dressings are indeed adhesive and often fall off in daily application, so the adhesive ability required for ideal dressings can be reasonably realized by using the diverse functions of functional hydrogel dressings [144]. The hydrogel with excellent adhesion can prevent bacterial invasion and inhibit its proliferation on wounds [145].

**Figure 22:**
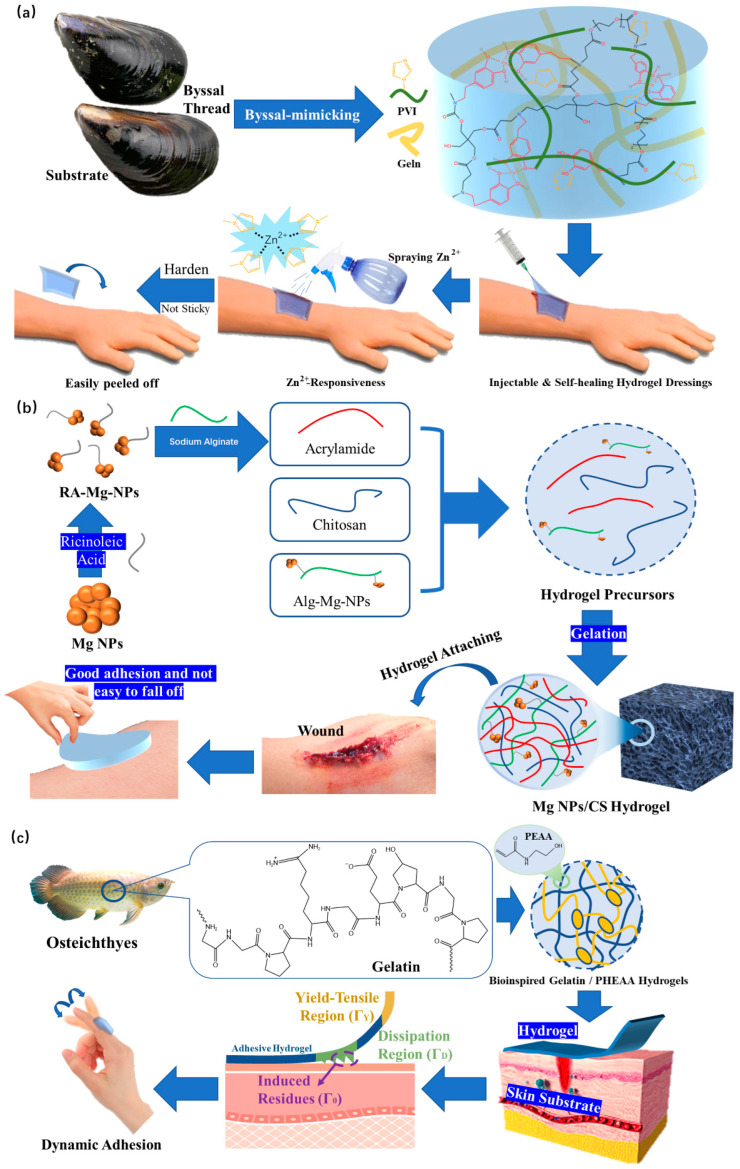
(**a**) The design strategy of the HB-PBAE/Geln/PVI composite hydrogel dressing and the mechanism of its special removal method of dynamic dressing. (**b**) Preparation of the MgNPs/CS hydrogel dressing and its application in wound healing. (**c**) Design strategy and dynamic adhesion mechanism of gelatin/PHEAA composite hydrogel dressings.

The team of Professor Liu and Professor Wang [146] developed a new hydrogel dressing based on supramolecular and metal ion coordination. As an ideal adhesive, dopamine (DOPA) can provide excellent wound adhesion properties for dressings. Fe^3+^ can form coordination bonds with DOPA to stabilize hydrogels, producing hydrogel dressings with strong adhesion. When the dressings need to be replaced, a Zn^2+^ aqueous solution simply needs to be sprayed on the dressing, and the adhesion ability of the hydrogel will be significantly reduced. At the same time, poly (1-vinylimidazole) (PVI) and Zn^2+^ form stable coordination bonds, further enhancing the strength of the hydrogel, so that the dressing can be easily replaced without damage, and there will be no obvious dressing residue at the site of the wound (Figure 22a). This is a scheme that can improve adhesion and avoid secondary injury.

In order to avoid secondary injury, there are other design ideas that can use excellent biodegradability to directly avoid the manual removal of dressings. Qu [147] developed a hydrogel composed of chitosan (CS), polyacrylamide, etc., in which MgNPs were introduced. They successfully prepared a multi-mesh nanocomposite MgNPs/CS hydrogel dressing. The MgNPs/CS composite hydrogel dressing showed strong adhesion to skin in performance tests (Figure 22b). Not only can nonbiological materials improve adhesion, but biological materials can also achieve this goal. Wang et al. [148] successfully prepared a gelatin/PHEAA hydrogel dressing after loading a hydrogel prepared by acrylamide with gelatin from fish (Figure 22c). This product can adapt to the wounds located in areas with high-frequency movement. Tests were performed using different concentrations of gelatin, and it was found that in order applications in human therapy, hydrogel dressings with concentrations of gelatin between 0.1 and 0.2 g/mL had the best adhesion to the wound. The controllable mediating function of the adhesion of the hydrogel dressing is realized. Other research results show that compared with nonbiological materials, biological materials can not only improve adhesion, but can also avoid the step of removing dressings, thus essentially avoiding secondary injury. Both gelatin [149] extracted from fish and MgNP [150] are environmentally friendly materials that are low-cost and easy to obtain, in addition to their excellent biodegradability. This also prevents secondary damage caused by high viscosity when removing the dressings.

#### 4.3.2. Extensibility

**Figure 23:**
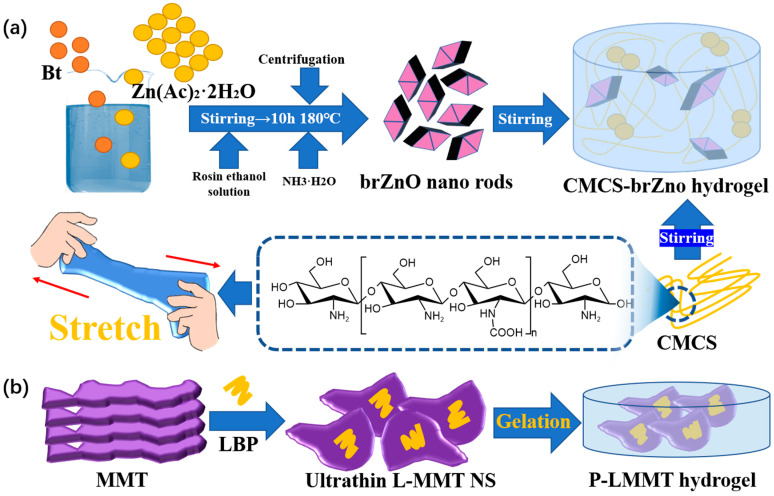
(**a**) The synthesis process of CMCS-brZnO hydrogel and the schematic diagram showing its strong tensile properties. (**b**) Preparation diagram of P-L-MMT hydrogel.

In terms of tensile properties [151], the addition of metal oxides is a common method to improve the function of hydrogel dressings [152]. Hu et al. [153] synthesized fusiform zinc oxide nanorods (brZnO) using the hydrothermal method (Figure 23a). A carboxyl chitosan CMCS zinc bromide oxide composite hydrogel dressing was prepared. In it, brZnO increases the mechanical properties of the gel networks through the supramolecular interactions between brZnO and -OH, -COOH, and -NH. The higher the content of brZnO is, the higher the crosslinking density of the hydrogel is. Thus, the compact network structure with enhanced mechanical strength can retain more water without breaking [154], so as to better fit skin wounds.

In addition, the use of aluminosilicate is another common method to improve the tensile properties of hydrogel dressings. Using *Lycium barbarum* L. polysaccharides (LBPs), montmorillonite (MMT), and polyvinyl alcohol (PVA), Shi et al. [155] invented a composite hydrogel dressing with LBP-functionalized montmorillonite nanosheets (L-MMT NS) (Figure 23b). The P-L-MMT hydrogel dressing has excellent swelling performance, which is conducive to blood adsorption and removal of wound metabolites and promotes the healing of bleeding wounds. At the same time, the tensile strength of P-L-MMT hydrogel is still higher than that of human skin. It shows that P-L-MMT hydrogel has good flexibility.

## 5. Summary and Prospects

The recent progress in the preparation of hydrogel dressings with various functions conducive to wound repair were highlighted in this review. Functional hydrogel dressings have become products with excellent therapeutic impacts as a result of ongoing exploration into their development. Especially in recent years, related research on how to make the function of hydrogels more conducive to wound repair has resulted in many breakthroughs, including dressings with injectability, self-healing capabilities, controlled drug release, healing promotion, antibacterial activity, hemostasis effects, and improved mechanical properties. Furthermore, many future prospects can be summarized as follows.

How to ensure hydrogel dressings can quickly self-repair and adapt to a wound by reversible covalent bonds and noncovalent bonds has always been an important research question. In this review, we found that borate bonds are generally superior to imine bonds, hydrogen bonds, and metal coordination bonds. Faced with the need to shorten their adaptive and self-healing times, combining reversible covalent bonds and noncovalent bonds in hydrogel dressings to synergistically improve related functions is one potential focus challenge of future research.

Another significant aspect of functional hydrogel dressings is the controlled release of drugs. These dressings can be designed to respond to different signals, such as pH, ROS, glucose, pressure, and temperature, so as to accurately control the release of drugs into the wound. When treating different wounds, research on how to construct a system that can control drug release via environmental changes and human subjective factors to accelerate wound healing will be indispensable in the future. It is also worth noting that new cells are introduced into tissues through functional hydrogel dressings to achieve wound self-repair [156,157]. This is a promising and emerging route in the field of hydrogel dressings, where the dressings act as controlled release systems for delivering cells to the wound site to promote tissue regeneration. However, further research and development in this area is still needed for future investigations in the field of hydrogel dressings.

Meanwhile, in order to create an environment conducive to wound repair, the recent progress of functional hydrogel dressings loaded with chemical particles with different functions was highlighted. Surprisingly, we found that hydrogel dressings containing chemical particles can successfully create an ideal environment for wound repair. However, most of the chemical particles employed today are hazardous and expensive, and extended use causes some harm to the human body. Therefore, it is necessary to find more and better chemical particles suitable for wound repair with lower costs. This will directly determine the evaluation results of functional hydrogel dressings in terms of their ability to create a therapeutic environment and would thus be a fruitful area for further work. Furthermore, wounds comprise a dynamic microenvironment that changes throughout the healing process. Presently, most hydrogel dressings with antibacterial properties focus on the overall healing process and antibacterial impact, without considering the timing and effectiveness of the dressings during different stages of healing. Moving forward, our research will concentrate on developing antibacterial hydrogel wound dressings that can precisely control the duration of antibacterial activity. This represents a highly promising research direction for functional hydrogels in the field of wound care.

Based on a market report [158], functional hydrogel dressings have witnessed a growing adoption rate in clinical wound treatment, holding the largest revenue share in the chronic wound repair market since 2021. These dressings have shown remarkable therapeutic effects for difficult-to-heal chronic wounds. In recent years, there has been a trend towards reducing raw material costs in both commercialized products and research findings, leading to increased interest in the commercialization of high-end dressing products.

As a result, it is predicted that future research on functional hydrogel dressings will focus on cost reduction and improved specificity for commercial applications, while continuing to advance research in acute wound repair. The treatment of chronic wounds with functional hydrogel dressings will be a major research focus moving forward. To achieve this, the combinations of preparation methods discussed in this review and exploration of new methods will be necessary to develop more functional hydrogel dressings. Enhancing the functionality of dressings, optimizing preparation methods, and selecting cost-effective raw materials will remain significant challenges for future research and problem-solving efforts.

An large body of literature has been published that emphasizes the value of functional hydrogel dressings. It would be very beneficial for wound healing if more research was conducted in this area. Many efforts are still needed in the following areas: (1) the acceleration of wound adaptation and self-healing; (2) drug selection and reasonable controlled release systems; (3) enhancing antibacterial safety and ability; (4) shortening time to reach hemostasis; and (5) improving the mechanical properties of hydrogels. This review has the potential to enhance not just the meaning of wound healing but also the reach of theoretical studies in chemistry, clinical medicine, and materials science. When developing and innovating new functional hydrogel wound dressings, this review can offer new ideas and mechanisms for researchers in the future, advancing the field of wound dressing therapy and expanding the market for functional hydrogel dressings.

## Figures and Tables

**Table 1:** The main materials, advantages, and relevant performance parameters of reversible covalent bond hydrogel dressings.

Hydrogel Components	Dynamic Bond	Self-Healing Time	Injectable	Advantages	Ref.
OMCC from PP+ CMCS from HER	Imine bond	5 h	YES	Non-toxicity and biosecurity	[47]
AHA + ADA + oxi-CNC	Imine bond	4 h	YES	Improves hydrogels’ strength without sacrificing their self-healing property	[49]
CS + COL	Imine bond	45 min	YES	DA-PEG allows the chain between the two crosslinking points to remain flexible	[50]
OSA-DA + PAM	Imine bond	15 min	NO	Ultratough and self-healing	[46]
HA-ADH + PF127-CHO	Imine bond	10 min	YES	The use of biological materials greatly improves the performance of the dressing	[52]
HPC + Phenylboronic Acid	Borate ester bond	10 min	YES	Blood compatibility, anti-oxidation, good tissue adhesion	[59]
boronic acid + catechol groups	Borate ester bond	5 min	YES	Treats wounds with high microbial bioburdens	[58]
CS + OHA	Imine bond	2 min	YES	Self-heals quickly and its materials have anti-inflammatory effects	[53]
OCMC-DA + PVA + CNF	Borate ester bond	12 s	YES	Ultrastretchable and simple preparation method	[57]
Catechin of PDA + Phenylboric Acid	Borate ester bond	Immediately	YES	Has the fastest self-healing speed	[60]

**Table 2:** The modified hydrogel dressings and their stimuli-responsive components, response signals, and advantages.

Stimuli	Stimuli Responsive Component	Advantage	Ref.
pH	Cystamine	Drug delivery carrier, can recognize acidic or reducing conditions	[78]
	Oligonucleotides rich in adenine and cytosine	Biocompatibility and biodegradability	[79]
ROS	Thiol-maleimide	Protected encapsulated mesenchymal stem cells from cytotoxic doses of ROS	[80]
	Diclofenac sodium and mangiferin	Eliminate oxygen free radicals, inhibit inflammatory reaction and generate new skin	[81]
	L-Arginine	It has the ability of absorbing wound exudates and controlling drug release	[82]
Temperature	Chitosan and solubilized placental extracellular matrix	Rapid response to temperature changes	[83]
	Muco-mimetic poloxamer 407 (F-107) and TS-Gel-Ag-col	Prevent gel from being diluted in contact with blood	[84]
Glucose	Polyvinyl alcohol and chitosan grafted with phenylboric acid	The drug is released as needed according to the glucose concentration	[85]
	Glucose oxidase and DG@Gel	Breaks down excess glucose	[86]
Pressure	Conductive fillers	Secondary injury caused by unconscious physical movement	[87]
	F127DA micelles	Wounds on frequently moving body parts can be effectively treated	[88]

**Table 3:** Typical metals or metal compounds used to enhance the antibacterial activity of hydrogel dressings.

Name	Classification	Component	Advantages	Refs
Nano-silver hydrogel	Nanoparticles	Ag-NPs	Maintains antibacterial efficacy	[114]
PVA/CMCS/AgNPs/Borax hydrogel	Nanoparticles	Ag-NPs	1. Extended antibacterial activity 2. Increased mechanical strength	[115]
PAA-AuNPs hydrogel	Nanoparticles	Au-NPs	1. Improved antibacterial activity of drugs 2. Activates drug precursors	[116]
PSNZn composite hydrogel	Metal ions	PDA/PSBMA/NFC/Zn^2+^	1. Antibacterial property 2. Hemostasis	[117]
Ag@ZnO NCs impregnated hydrogels	Nanoparticles	PVP/PVA/Ag@ZnO	1. Antibacterial 2. Anti-biofilm activity	[118]
Silver-sulfadiazine-loaded antibacterial hydrogel	Sulfa antibiotics with metal	Silver sulfadiazine	1. Antiresistant 2. Antibacterial	[119]

## Data Availability

No data were used for the research described in the article.

## Abbreviations

**ADA:** hydrazide-modified sodium hyaluronate; **AHA:** aldehyde-modified sodium hyaluronate; **APS:** ammonium persulfate; **AM:** acrylamide; **A:** adenine; **BIS:** N,N′-methylenebis-acrylamide; **brZnO:** zinc oxide nanorods; **CMCS:** carboxymethyl chitosan; **COL:** Collagen; **CNF:** cellulose nanofibers, **CA:** cystamine; **C:** cytosine; **CHX:** chlorhexidine diacetate; **CLNs:** CHX-loaded nanogels; **CS:** chitosan; **CMCS:** carboxymethyl chitosan; **DA:** dopamine hydrochloride; **dop:** dopamine; **DFO:** desferrioxamine; **DOPA:** dopamine; **EGCG:** epigallocatechin-3-gallate; **EDC:** 1-ethyl-3-[3-(dimethylamino)propyl]carbodiimide; **Fmoc-F:** Fluorenylmethyloxycarbonyl-phenylalanine; **GC:** glycol chitosan; **GA:** gallic acid; **HER:** hericium erinaceus residues; **HA-ADH:** hyaluronate-adipic dihydrazide; **HPC:** hydroxypropyl chitosan; **HisMA:** histidine methacrylamide; **HA:** hyaluronic acid; **HNTs:** halloysite nanotubes; **KA:** kaolin; **L-Arg:** L-arginine; **LAP:** laponite; **LBP:** Lycium barbarum L. polysaccharide; **L-MMT NS:** LBP-functionalized montmorillonite nanosheets; **MY:** myricetin; **mPEG-MA:** methacrylated methoxy polyethylene glycol; **MgNPs:** Mg(OH)_2_ nanoparticles; **MMT:** montmorillonite; **NHS:** N-Hydroxy succinimide; **NIPAAm:** N-isopropylacrylamide; **nWH:** nano whitlockite; **OSA-DA:** dopamine-grafted oxidized sodium alginate; **OMCC:** oxidized microcrystalline cellulose; **oxi-CNC:** aldehyde-modified cellulose nanocrystals; **OHA:** oxidized hyaluronic acid; **OCMC-DA:** dopamine-grafted oxidized carboxymethyl cellulose; **OHAdop:** catechol-modified oxidized hyaluronic acid; **PP:** pineapple peel; **PB:** polyol/borax; **PDA:** polydopamine; **PAHC:** phenylboric acid-modified hydroxypropyl cellulose; **PA:** protocatechualdehyde; **P(AM-HisMA):** poly(acrylamide-histidine methacrylamide); **PAM:** polyacrylamide; **PVP:** polyvinylpyrrolidone; **PBS:** Phosphate-buffered saline; **PEGDE:** poly(ethylene glycol) diglycidyl ether; **PEG:** Poly(ethylene glycol); **PTK:** polythioketal; **PEG-DA:** polyethylene glycol diacrylates; **PBA:** phenylboronic acid; **pNIPAm:** poly(N-isopropylacrylamide); **poly(Cu-NIPAm):** poly(Cu-N-isopropylacrylamide); **PVA:** polyvinyl alcohol; **PAAm:** polyacrylamide; **PHEAA:** poly(N-hydroxyethyl acrylamide); **PVA:** polyvinyl alcohol; **QCS:** quaternized chitosan; **ROS:** reactive oxygen species; **SH-PEG:** thiolated polyethylene glycol; **SA:** sodium alginate; **TA:** tannic acid.

## References

[1] Liu S.D., Li D., Wang Y., Zhou G.Q., Ge K., Jiang L. (2023). Adhesive, antibacterial and double crosslinked carboxylated polyvinyl alcohol/chitosan hydrogel to enhance dynamic skin wound healing. Int. J. Biol. Macromol..

[2] QYResearch (2021). 2021–2027 Global and China Advanced Dressing Market Status and Forecast. https://www.qyresearch.com.cn/reports/2021-2027-p911142.html.

[3] Tang N.F.R., Heryanto H., Armynah B., Tahir D. (2023). Bibliometric analysis of the use of calcium alginate for wound dressing applications: A review. Int. J. Biol. Macromol..

[4] Aycan D., Selmi B., Kelel E., Yildirim T., Alemdar N. (2019). Conductive polymeric film loaded with ibuprofen as a wound dressing material. Eur. Polym. J..

[5] Ajiteru O., Lee O.J., Kim J.H., Lee Y.J., Lee J.S., Lee H., Sultan M.T., Park C.H. (2022). Fabrication and characterization of a myrrh hydrocolloid dressing for dermal wound healing. Colloids Interface Sci. Commun..

[6] Tang S., Gong Z.J., Wang Z.F., Gao X., Zhang X.N. (2022). Multifunctional hydrogels for wound dressings using xanthan gum and polyacrylamide. Int. J. Biol. Macromol..

[7] Yang Y., Ren Y.Q., Song W., Yu B.H., Liu H.Z. (2022). Rational design in functional hydrogels towards biotherapeutics. Mater. Des..

[8] Van Tomme S.R., Storm G., Hennink W.E. (2008). In situ gelling hydrogels for pharmaceutical and biomedical applications. Int. J. Pharm..

[9] QYResearch (2022). 2022–2026 Global and China Medical Grade Hydrogel Market Status and Forecast. https://www.qyresearch.com.cn/reports/medical-grade-hydrogel-p978747.html.

[10] Elangwe C.N., Morozkina S.N., Olekhnovich R.O., Krasichkov A., Polyakova V.O., Uspenskaya M.V. (2022). A Review on Chitosan and Cellulose Hydrogels for Wound Dressings. Polymers.

[11] Winter G.D. (1962). Formation of the scab and the rate of epithelization of superficial wounds in the skin of the young domestic pig. Nature.

[12] Wang L.R., Zhou M.Y., Xu T.L., Zhang X.J. (2022). Multifunctional hydrogel as wound dressing for intelligent wound monitoring. Chem. Eng. J..

[13] Wang T., Yi W.W., Zhang Y., Wu H., Fan H.W., Zhao J.L., Wang S.G. (2023). Sodium alginate hydrogel containing platelet-rich plasma for wound healing. Colloids Surf. B.

[14] Li M., Liang Y.P., He J.H., Zhang H.L., Guo B.L. (2020). Two-Pronged Strategy of Biomechanically Active and Biochemically Multifunctional Hydrogel Wound. Chem. Mater..

[15] Huang X.X., Ma C., Xu Y.C., Cao J.F., Li J.C., Li J.Z., Shi S.Q., Gao Q. (2022). A tannin-functionalized soy protein-based adhesive hydrogel as a wound dressing. Ind. Crop. Prod..

[16] Zeng Z., Zhu M., Chen L., Zhang Y., Lu T., Deng Y., Ma W., Xu J., Huang C., Xiong R. (2022). Design the molecule structures to achieve functional advantages of hydrogel wound dressings: Advances and strategies. Compos. B. Eng..

[17] Nazarnezhada S., Abbaszadeh-Goudarzi G., Samadian H., Khaksari M., Ghatar J.M., Khastar H., Rezaei N., Mousavi S.R., Shirian S., Salehi M. (2020). Alginate hydrogel containing hydrogen sulfide as the functional wound dressing material: In vitro and in vivo study. Int. J. Biol. Macromol..

[18] Guo S., Dipietro L.A. (2010). Factors affecting wound healing. J. Dent. Res..

[19] Zhu J.Y., Zhong K.J., Zong Y., Wang S.H., Yang H.Y., Zhen L., Tao S.Y., Sun L.Z., Yang J.J., Li J.Y. (2022). A mussel-inspired wet-adhesion hydrogel with hemostasis and local anti-inflammation for managing the development of acute wounds. Mater. Des..

[20] Zeng H.H., Liu X., Zhang Z.Q., Song X.W., Quan J., Zheng J., Shen Z.L., Ni Y.Q., Liu C.T., Zhang Y. (2022). Self-healing, injectable hydrogel based on dual dynamic covalent cross-linking against postoperative abdominal cavity adhesion. Acta Biomater..

[21] Liu Z.B., Li J., Zhang Z.P., Liu J.Z., Wu C.Y., Yu Y.Q. (2023). Incorporating self-healing capability in temperature-sensitive hydrogels by non-covalent chitosan crosslinkers. Eur. Polym. J..

[22] Erezuma I., Lukin I., Desimone M., Zhang Y.S., Dolatshahi-Pirouz A., Orive G. (2023). Progress in self-healing hydrogels and their applications in bone tissue engineering. Biomater. Adv..

[23] Karvinen J., Kellomäki M. (2022). Characterization of self-healing hydrogels for biomedical applications. Eur. Polym. J..

[24] Lv J.H., Fang Y.R., Wu M., Ou X.Y., Zhang W.C., Wang S.Y., Li H.G., Shang L., He M.F., Zhao Y. (2023). Poly(acrylamide) hydrogel composites with microsized β-chitin fiber and the properties of mechanical and drug release. Mater. Today Commun..

[25] Wang Z.G., Chen R.P., Yang S.P., Li S., Gao Z.X. (2022). Design and application of stimuli-responsive DNA hydrogels: A review. Mater. Today Bio.

[26] He W.Y., Wang X.C., Gong W., Huang H.B., Hou Y.Y., Wang R., Hu J.N. (2023). Construction of an antibacterial hydrogel based on diammonium glycyrrhizinate and gallic acid for bacterial-infected wound healing. Colloids Surf. B.

[27] Yao H., Wu M., Lin L.W., Wu Z.L., Bae M.J., Park S.M., Wang S.L., Zhang W., Gao J.F., Wang D.G. (2022). Design strategies for adhesive hydrogels with natural antibacterial agents as wound dressings: Status and trends. Mater. Today Bio..

[28] Chang R., Zhao D.H., Zhang C., Liu K.Y., He Y.M., Guan F.X., Yao M.H. (2023). Nanocomposite multifunctional hyaluronic acid hydrogel with photothermal antibacterial and antioxidant properties for infected wound healing. Int. J. Biol. Macromol..

[29] Luo W., Hu B., Zhang H.L., Li C.Y., Shi Y.P., Li X.C., Jin L. (2023). Antibacterial photothermal and stable Ag-titanium-oxo-clusters hydrogel designed for wound healing. Mater. Des..

[30] Cao S.J., Bi Z.J., Li Q.J., Zhang S.K., Singh M., Chen J.D. (2023). Shape memory and antibacterial chitosan-based cryogel with hemostasis and skin wound repair. Carbohydr. Polym..

[31] Takeno H., Kimura Y. (2016). Molecularweight effects on tensile properties of blend hydrogels composed of clay and polymers. Polymer.

[32] Wang W.D., Ummartyotin S., Narain R. (2023). Advances and Challenges on Hydrogels for Wound Dressing. Curr. Opin. Biomed. Eng..

[33] Ding X.Y., Yu Y.R., Zu Y. (2023). Self-healing hydrogels based on the Knoevenagel condensation reaction for wound healing. Biomed. Technol..

[34] Deng Z.X., Guo Y., Zhao X., Ma P.X., Guo B.L. (2018). Multifunctional stimuli-responsive hydrogels with self-Healing, high conductivity, and rapid recovery through host–guest interactions. Chem. Mater..

[35] Han W., Chen C., Yang K., Wang H.B., Xia H.G., Zhao Y., Teng Y., Feng G.C., Chen Y.M. (2023). Hyaluronic acid and chitosan-based injectable and self-healing hydrogel with inherent antibacterial and antioxidant bioactivities. Int. J. Biol. Macromol..

[36] Gao G.R., Yang F.J., Zhou F.H., He J., Lu W.I., Xiao P., Yan H.Z., Pan C.F., Chen T., Wang Z.L. (2020). Bioinspired self-healing human-machine interactive touch pad with pressure-sensitive adhesiveness on targeted substrates. Adv. Mater..

[37] Wang Z.Y., Gu J.Y., Zhang D.F., Zhang Y., Chen J.H. (2023). Structurally dynamic gelatin-based hydrogels with self-healing, shape memory, and cytocompatible properties for 4D printing. Biomacromolecules.

[38] Zhang Z., Abidi N., Lucia L.A. (2022). Dual crosslinked-network self-healing composite hydrogels exhibit enhanced water adaptivity and reinforcement. Ind. Eng. Chem. Res..

[39] Feng W.J., Wang Z.K. (2022). Shear-thinning and self-healing chitosan-graphene oxide hydrogel for hemostasis and wound healing. Carbohydr. Polym..

[40] Zhang K.Y., Feng Q., Fang Z.W., Gu L., Bian L.M. (2021). Structurally dynamic hydrogels for biomedical applications: Pursuing a fine balance between macroscopic stability and microscopic dynamics. Chem. Rev..

[41] Cromwell O.R., Chung J., Guan Z.B. (2015). Malleable and self-Healing covalent polymer networks through tunable dynamic boronic ester bonds. J. Am. Chem. Soc..

[42] Ikura R., Park J., Osaki M., Yamaguchi H., Harada A., Takashima Y. (2022). Design of self-healing and self-restoring materials utilizing reversible and movable crosslinks. NPG Asia Mater..

[43] Schiff H. (1864). Mittheilungen aus dem Universitätslaboratorium in Pisa: Untersuchungen ü das Chinolin. Justus Liebigs Ann. Chem..

[44] Pogostin B.H., Saenz G., Cole C.C., Euliano E.M., Hartgerink J.D., McHugh K.J. (2023). Dynamic imine bonding facilitates mannan release from a nanofibrous peptide hydrogel. Bioconjug. Chem..

[45] Min J.B., Zhou Z.X., Wang H.N., Chen Q.H., Hong M.C., Fu H.Q. (2022). Room temperature self-healing and recyclable conductive composites for flexible electronic devices based on imine reversible covalent bond. J. Alloys Compd..

[46] Chen T., Chen Y.J., Rehman H.U., Chen Z., Yang Z., Wang M., Li H., Liu H.Z. (2018). Ultratough self-healing and tissue-adhesive hydrogel for wound dressing. ACS Appl. Mater. Interfaces.

[47] Yin H.S., Song P.Q., Chen X.Y., Huang Q.Y., Huang H.H. (2022). A self-healing hydrogel based on oxidized microcrystalline cellulose and carboxymethyl chitosan as wound dressing material. Int. J. Biol. Macromol..

[48] Nie L., Wei Q.Q., Sun M., Ding P., Wang L., Sun Y.F., Ding X.Y., Okoro O.V., Jiang G.H., Shavandi A. (2023). Injectable, self-healing, transparent, and antibacterial hydrogels based on chitosan and dextran for wound dressings. Int. J. Biol. Macromol..

[49] Li S.Z., Dong Q., Peng X.T., Chen Y., Yang H.J., Xu W.L., Zhao Y.T., Xiao P., Zhou Y.S. (2022). Self-healing hyaluronic acid nanocomposite hydrogels with platelet-rich plasma impregnated for skin regeneration. ACS Nano.

[50] Ding C.C., Tian M.D., Feng R., Dang Y., Zhang M. (2020). Novel self-healing hydrogel with injectable, pH-responsive, strain-sensitive, promoting wound-healing, and hemostatic properties based on collagen and chitosan. ACS Biomater. Sci. Eng..

[51] Zhang X., Tan B.W., Wu Y.T., Zhang M., Xie X., Liao J.F. (2022). An injectable, self-healing carboxymethylated chitosan hydrogel with mild photothermal stimulation for wound healing. Carbohydr. Polym..

[52] Mei L., Zhang D.J., Shao H.R., Hao Y.P., Zhang T., Zheng W.P., Ji Y.J., Ling P.X., Lu Y., Zhou Q.H. (2022). Injectable and self-healing probiotics-loaded hydrogel for promoting superbacteria-infected wound healing. ACS Appl. Mater. Interfaces.

[53] Liu S.X., Jiang N., Chi Y.Q., Peng Q., Dai G.R., Qian L., Xu K.M., Zhong W.Y., Yue W.Q. (2022). Injectable and self-Healing hydrogel based on chitosan-tannic acid and oxidized hyaluronic acid for wound healing. ACS Biomater. Sci. Eng..

[54] Chen M.T., Wu Y., Chen B.H., Tucker A.M., Jagota A., Yang S. (2022). Fast, strong, and reversible adhesives with dynamic covalent bonds for potential use in wound dressing. Proc. Natl. Acad. Sci. USA.

[55] Ivanov A.E., Larsson H., Galaev I.Y., Mattiasson B. (2004). Synthesis of boronate-containing copolymers of *N*,*N*-dimethylacrylamide, their interaction with poly(vinyl alcohol) and rheological behaviour of the gels. Polymer.

[56] Chen Y.M., Qian W.Q., Chen R., Zhang H.J., Li X.J., Shi D.J., Dong W.F., Chen M.Q., Zhao Y. (2017). One-pot preparation of autonomously self-healable elastomeric hydrogel from boricacid and random copolymer bearing hydroxyl groups. ACS Macro Lett..

[57] Zhong Y.J., Seidi F., Li C.C., Wan Z.M., Jin Y.C., Song J.L., Xiao H.N. (2021). Antimicrobial/biocompatible hydrogels dual-reinforced by cellulose as ultrastretchable and rapid self-Healing wound dressing. Biomacromolecules.

[58] Zhong Y.J., Seidi F., Wang Y.L., Zheng L., Jin Y.C., Xiao H.N. (2022). Injectable chitosan hydrogels tailored with antibacterial and antioxidant dual functions for regenerative wound healing. Carbohydr. Polym..

[59] Deng P.P., Chen F.X., Zhang H.D., Chen Y., Zhou J.P. (2021). Conductive, Self-Healing, adhesive, and antibacterial hydrogels based on lignin/cellulose for rapid MRSA-infected wound repairing. ACS Appl. Mater. Interfaces.

[60] Deng P.P., Liang X., Chen F.X., Chen Y., Zhou J.P. (2022). Novel multifunctional dual-dynamic-bonds crosslinked hydrogels for multi-strategy therapy of MRSA-infected wounds. Appl. Mater. Today.

[61] Xue K., Liow S.S., Karim A.A., Li Z.B., Loh X.J. (2018). A recent perspective on noncovalently formed polymeric hydrogels. Chem. Rec..

[62] Hou B.N., Shen H.L., Li J., Xie W.Q., Li Z.Z. (2020). Self-healing polymer hydrogel based on dynamic chemical bonds. J. Mater. Eng..

[63] Qian J.M., Ji L.J., Xu W.J., Hou G.H., Wang J.L., Wang Y.P., Wang T.B. (2022). Copper-hydrazide coordinated multifunctional hyaluronan hydrogels for infected wound healing. ACS Appl. Mater. Interfaces.

[64] Rather R.A., Sarwara R.K., Das N., Pal B. (2019). Impact of reducing and capping agents on carbohydrates for the growth of Ag and Cu nanostructures and their antibacterial activities. Particuology.

[65] Chen H., Cheng R.Y., Zhao X., Zhang Y.H., Tam A., Yan Y.F., Shen H.K., Zhang Y.S., Qi J., Feng Y.H. (2019). An injectable self-healing coordinative hydrogel with antibacterial and angiogenic properties for diabetic skin wound repair. NPG Asia Mater..

[66] Hu J.L., Feng K.K., Cong Y.Y., Li X.Y., Jiang Y.J., Jiao X.D., Li Y.R., Zhang Y.Q., Dong X.Y., Lu W.F. (2022). Nanosized shikonin-Fe(III) coordination material for synergistic wound treatment: An initial explorative study. ACS Appl. Mater. Interfaces.

[67] Liang Y.Q., Li Z.L., Huang Y., Yu R., Guo B.L. (2021). Dual-dynamic-bond cross-linked antibacterial adhesive hydrogel sealants with on-demand removability for post-wound-closure and infected wound healing. ACS Nano.

[68] Zhang H., He J.D., Qu J.Q. (2022). Metal-coordinated amino acid hydrogels with ultra-stretchability, adhesion, and self-healing properties for wound healing. Eur. Polym. J..

[69] Devi VK A., Shyam R., Palaniappan A., Jaiswal A.K., Oh T.-H., Nathanael A.J. (2021). Self-Healing Hydrogels: Preparation, Mechanism and Advancement in Biomedical Applications. Polymers.

[70] Zhao D.W., Feng M., Zhang L., He B., Chen X.Y., Sun J. (2021). Facile synthesis of self-healing and layered sodium alginate/polyacrylamide hydrogel promoted by dynamic hydrogen bond. Carbohydr. Polym..

[71] Yu H., Xiao Q.H., Qi G.L., Chen F.X., Tu B.Y., Zhang S., Li Y.P., Chen Y., Yu H., Duan P. (2022). A hydrogen bonds-crosslinked hydrogels with self-healing and adhesive properties for hemostatic. Front. Bioeng. Biotechnol..

[72] Guo H.L., Huang S., Xu A., Xue W. (2022). Injectable adhesive self-healing multiple-dynamic-bond crosslinked hydrogel with photothermal antibacterial activity for infected wound healing. Chem. Mater..

[73] Ying R., Huang W.C., Mao X.Z. (2022). Synthesis of agarose-based multistimuli-responsive hydrogel dressing for accelerated wound healing. ACS Biomater. Sci. Eng..

[74] Feng S.M., Wang L.L., Shao P., Sun P.L., Yang C.S. (2022). A review on chemical and physical modifications of phytosterols and their influence on bioavailability and safety. Crit. Rev. Food Sci. Nutr..

[75] Xu K.K., Yao H., Fan D., Zhou L., Wei S.H. (2021). Hyaluronic acid thiol modified injectable hydrogel: Synthesis, characterization, drug release, cellular drug uptake and anticancer activity. Carbohydr. Polym..

[76] Siepmann J., Siepmann F. (2008). Mathematical modeling of drug delivery. Int. J. Pharm..

[77] Zelikin A.N., Ehrhardt C., Healy A.M. (2016). Materials and methods for delivery of biological drugs. Nat. Chem..

[78] Komatsu S., Tago M., Ando Y., Asoh T.A., Kikuchi A. (2021). Facile preparation of multi-stimuli-responsive degradable hydrogels for protein loading and release. J. Control. Release.

[79] Hu Y.W., Gao S.J., Lu H.F., Ying J.Y. (2022). Acid-resistant and physiological pH-responsive DNA hydrogel composed of A-motif and i-motif toward oral insulin delivery. J. Am. Chem. Soc..

[80] Martin J.R., Patil P., Yu F., Gupta M.K., Duvall C.L. (2020). Enhanced stem cell retention and antioxidative protection with injectable, ROS-degradable PEG hydrogels. Biomaterials.

[81] Wu Y., Wang Y., Long L.Y., Hu C., Kong Q.Q., Wang Y.B. (2022). A spatiotemporal release platform based on pH/ROS stimuli-responsive hydrogel in wound repairing. J. Control. Release.

[82] Yu J., Zhang R.L., Chen B.H., Liu X.L., Jia Q., Wang X.F., Yang Z., Ning P.B., Wang Z.L., Yang Y. (2022). Injectable reactive oxygen species-responsive hydrogel dressing with sustained nitric oxide release for bacterial ablation and wound healing. Adv. Funct. Mater..

[83] Azadbakht A., Alizadeh S., Ahovan Z.A., Khosrowpour Z., Majidi M., Pakzad S., Shojaei S., Chauhan N.P.S., Jafari M., Gholipourmalekabadi M. (2022). Chitosan-placental ECM composite thermos-responsive hydrogel as a biomimetic wound dressing with angiogenic property. Macromol. Biosci..

[84] Liu X.H., Hou M.D., Luo X.M., Zheng M.H., Wang X.C., Zhang H.J., Guo J.L. (2021). Thermoresponsive hemostatic hydrogel with a biomimetic nanostructure constructed from aggregated collagen nanofibers. Biomacromolecules.

[85] Zhou W.Y., Duan Z.G., Fu J.Z.R.Z., Zhu C.H., Fan D.D. (2022). Glucose and MMP-9 dual-responsive hydrogel with temperature sensitive self-adaptive shape and controlled drug release accelerates diabetic wound healing. Bioact. Mater..

[86] Yang J.X., Zeng W.N., Xu P., Fu X.X., Yu X.J., Chen L., Leng F., Yu C., Yang Z.Y. (2022). Glucose-responsive multifunctional metal-organic drug-loaded hydrogel for diabetic wound healing. Acta Biomater..

[87] Li D.R., Fei X., Xu L.Q., Wang Y., Tian J., Li Y. (2022). Pressure-sensitive antibacterial hydrogel dressing for wound monitoring in bed ridden patients. J. Colloid Interface Sci..

[88] Fang K., Wang R., Zhang H., Zhou L.J., Xu T., Xiao Y., Zhou Y., Gao G.R., Chen J., Liu D.L. (2020). Mechano-responsive tough and antibacterial zwitterionic hydrogels with controllable drug release for wound healing applications. ACS Appl. Mater. Interfaces.

[89] Proksch E. (2018). pH in nature humans skin. Dermatol. J..

[90] Siegel R.A. (2014). Stimuli sensitive polymers and self regulated drug delivery systems: A very partial review. J. Control. Release.

[91] Cui T.T., Yu J.F., Wang C.F., Chen S., Li Q., Guo K., Qing R.K., Wang G.F., Ren J.N. (2022). Micro-gel ensembles for accelerated healing of chronic wound via pH regulation. Adv. Sci..

[92] Fan X.C., Yang L., Wang T., Sun T.D., Lu S.T. (2019). pH-responsive cellulose-based dual drug-loaded hydrogel for wound dressing. Eur. Polym. J..

[93] Yan Q., Liu L.L., Wang T., Wang H.N. (2019). A pH-responsive hydrogel system based on cellulose and dopamine with controlled hydrophobic drug delivery ability and long-term bacteriostatic property. Colloid Polym. Sci..

[94] Zhao H., Huang J., Li Y., Lv X.J., Zhou H.T., Wang H.R., Xu Y.Y., Wang C., Wang J., Liu Z. (2020). ROS-scavenging hydrogel to promote healing of bacteria infected diabetic wounds. Biomaterials.

[95] Li Y., Fu R.Z., Duan Z.G., Zhu C.H., Fan D.D. (2022). Injectable hydrogel based on defect-rich multi-nanozymes for diabetic wound healing via an oxygen self-supplying cascade reaction. Small.

[96] Pachua L., Mohammad F., Al-Lohedan H.A., Jawaid M. (2020). Chapter 5—Nanocellulose and nanohydrogel-mediated sustained drug delivery: Smart medical technology. Micro and Nano Technologies, Sustainable Nanocellulose and Nanohydrogels from Natural Sources.

[97] Wu M., Lu Z.H., Wu K.K., Nam C., Zhang L., Guo J.S. (2021). Recent advances in the development of nitric oxide-releasing biomaterials and their application potentials in chronic wound healing. J. Mater. Chem. B.

[98] Xu P.P., Zuo H.Q., Chen B., Wang R.J., Ahmed A., Hu Y., Ouyang J. (2017). Doxorubicin-loaded platelets as a smart drug delivery system: An improved therapy for lymphoma. Sci. Rep..

[99] Liang Y.P., Li M., Yang Y.T., Qiao L.P., Xu H.R., Guo B.L. (2022). pH/glucose dual responsive metformin release hydrogel dressings with adhesion and self-healing via dual-dynamic bonding for athletic dabetic foot wound healing. ACS Nano.

[100] Xu Z.J., Liu G.T., Huang J., Wu J. (2022). Novel Glucose-Responsive Antioxidant hybrid hydrogel for enhanced diabetic wound repair. ACS Appl. Mater. Interfaces.

[101] Hou M.D., Wang X.C., Yue O.Y., Zheng M.H., Zhang H.J., Liu X.H. (2022). Development of a multifunctional injectable temperature-sensitive gelatin-based adhesive double-network hydrogel. Biomater. Adv..

[102] FDA (2011). SOLARAZE® GEL. https://www.accessdata.fda.gov/drugsatfda_docs/label/2011/021005s013lbl.pdf.

[103] Horrocks A. (2006). Prontosan wound irrigation and gel: Management of chronic wounds. Br. J. Nurs..

[104] Rajati H., Alvandi H., Rahmatabadi S.S., Hosseinzadeh L., Arkan E. (2023). A nanofiber-hydrogel composite from green synthesized AgNPs embedded to PEBAX/PVA hydrogel and PA/Pistacia atlantica gum nanofiber for wound dressing. Int. J. Biol. Macromol..

[105] Zhang X.M., Liu Q., Zhu S.M., Yu M. (2022). Green and facile fabrication of nano-ZnO coated cellulose/starch/activated carbon hydrogel for enhanced dyes adsorption and antibacterial activity. Mater. Today Commun..

[106] Sundaram M.N., Amirthalingam S., Mony U., Varma P.K., Jayakumar R. (2019). Injectable chitosan-nano bioglass composite hemostatic hydrogel for effective bleeding control. Int. J. Biol. Macromol..

[107] Wang H., Lu Z.Y., Wang F.Y., Li Y.L., Ou Z.W., Jiang J.Y. (2023). A novel strategy to reinforce double network hydrogels with enhanced mechanical strength and swelling ratio by nano cement hydrates. Polymer.

[108] Thoniyot P., Tan M.J., Karim A.A., Young D.J., Loh X.J. (2015). Nanoparticle–hydrogel composites: Concept; design, and applications of these promising, multi-functional Materials. Adv. Sci..

[109] Wang A.H., Fan G.S., Qi H.L., Li H.Y., Pang C.C., Zhu Z.K., Ji S.C., Liang H., Jiang B.P., Shen X.C. (2022). H_2_O_2_-activated in situ polymerization of aniline derivative in hydrogel for real-time monitoring and inhibition of wound bacterial infection. Biomaterials.

[110] Zhou R., Zhou Q.X., Ling G.X., Zhang P. (2023). A cross-linked hydrogel of bismuth sulfide nanoparticles with excellent photothermal antibacterial and mechanical properties to combat bacterial infection and prompt wound healing. Colloids Surf. A Physicochem. Eng. Asp..

[111] Xiang J.X., Shen L., Hong Y.L. (2020). Status and future scope of hydrogels in wound healing: Synthesis, materials and evaluation. Eur. Polym. J..

[112] Shen J.F., Dai Y., Xia F., Zhang X.J. (2022). Role of divalent metal ions in the function and application of hydrogels. Prog. Polym. Sci..

[113] Elkhoury K., Morsink M., Sanchez-Gonzalez L., Kahn C., Tamayol A., Arab-Tehrany E. (2021). Biofabrication of natural hydrogels for cardiac, neural, and bone Tissue engineering Applications. Bioact. Mater..

[114] Song S., Liu Z., Abubaker M.A., Ding L., Zhang J., Yang S.R., Fan Z.J. (2021). Antibacterial polyvinyl alcohol/bacterial cellulose/nano-silver hydrogels that effectively promote wound healing. Mater. Sci. Eng. C.

[115] Liu Y.L., Mao J., Guo Z.Y., Hu Y.F., Wang S. (2022). Polyvinyl alcohol/carboxymethyl chitosan hydrogel loaded with silver nanoparticles exhibited antibacterial and self-healing properties. Int. J. Biol. Macromol..

[116] Mahmoud N.N., Hikmat S., Ghith D.A., Hajeer M., Hamadneh L., Qattan D., Khalil E.A. (2019). Gold nanoparticles loaded into polymeric hydrogel for wound healing in rats: Effect of nanoparticles’ shape and surface modification. Int. J. Pharm..

[117] Wang S.Y., Liu R.Q., Bi S.W., Zhao X.S., Zeng G.X., Li X.Y., Wang H.B., Gu J. (2022). Mussel-inspired adhesive zwitterionic composite hydrogel with antioxidant and antibacterial properties for wound healing. Colloids Surf. B Biointerfaces.

[118] Khan M.I., Paul P., Behera S.K., Jena B., Tripathy S.K., Lundborg C.S., Mishra A. (2021). To decipher the antibacterial mechanism and promotion of wound healing activity by hydrogels embedded with biogenic Ag@ZnO core-shell nanocomposites. Chem. Eng. J..

[119] McMahon S., Kennedy R., Duffy P., Vasquez J.M., Wall J.G., Tai H.Y., Wang W.X. (2016). Poly(ethylene glycol)-based hyperbranched polymer from RAFT and its application as a silver-sulfadiazine-loaded antibacterial hydrogel in wound care. ACS Appl. Mater. Interfaces.

[120] Gupta A., Mumtaz S., Li C.H., Hussain I., Rotello V.M. (2019). Combatting antibiotic-resistant bacteria using nanomaterials. Chem. Soc. Rev..

[121] Xie Y.Z.Y., Yang J., Zhang J.J., Zheng W.F., Jiang X.Y. (2020). Activating the antibacterial effect of 4,6-diamino-2-pyrimidinethiol-modified gold nanoparticles by reducing their sizes. Angew. Chem. Int. Ed..

[122] USGS (2022). Mineral Commodity Summaries 2022: U.S. Geological Survey.

[123] Kong Y., Hou Z.S., Zhou L.Q., Zhang P.F., Ouyang Y.W., Wang P.W., Chen Y.W., Luo X.L. (2021). Injectable self-healing hydrogels containing CuS nanoparticles with abilities of hemostasis, antibacterial activity, and promoting wound healing. ACS Biomater. Sci. Eng..

[124] Xia X.J., Liang Q.D., Sun X.G., Yu D.H., Huang X.R., Mugo S.M., Chen W., Wang D., Zhang Q., Conductive I.E. (2022). Antibacterial, and Anti-swelling Hydrogels as Implantable Sensors for Bioelectronics. Adv. Funct. Mater..

[125] Pohanka M. (2019). Copper and copper nanoparticles toxicity and their impact on basic functions in the body. Bratisl. Lek. Listy.

[126] Kircheva N., Dobrev S., Nikolova V., Angelova S., Dudev T. (2022). Theoretical insight into the phosphate-targeted silver’s antibacterial action: Differentiation between gram(+) and gram(−) bacteria. Inorg. Chem..

[127] Slavin Y.N., Asnis J., Häfeli U.O. (2017). Metal nanoparticles: Understanding the mechanisms behind antibacterial activity. J. Nanobiotechnol..

[128] Wang Y.M., Xiao D.D., Yu H.N., Ke R.Y., Shi S.L., Tang Y., Zhong Y., Zhang L.P., Sui X.F., Wang B.J. (2022). Asymmetric composite wound dressing with hydrophobic flexible bandage and tissue-adhesive hydrogel for joints skin wound healing. Compos. B Eng..

[129] Zhao X.Q., Wahid F., Zhao X.J., Wang F.P., Wang T.F., Xie Y.Y., Jia S.R., Zhong C. (2021). Fabrication of amino acid-based supramolecular hydrogel with silver ions for improved antibacterial properties. Mater. Lett..

[130] Marambio-Jones C., Hoek E.M.V. (2010). A review of the antibacterial effects of silver nanomaterials and potential implications for human health and the environment. J. Nanopart. Res..

[131] Vandebriel R.J., De Jong W.H. (2012). A review of mammalian toxicity of ZnO nanoparticles. Nanotechnol. Sci. Appl..

[132] Lohmann & Rauscher (2022). Suprasorb® A + Ag in the Treatment of Wounds at Risk of Infection and Infected Wounds. https://beta.clinicaltrials.gov/study/NCT05646121.

[133] Pourshahrestani S., Zeimaran E., Kadri N.A., Mutlu N., Boccaccini A.R. (2020). Polymeric hydrogel systems as emerging biomaterial platforms to enable hemostasis and wound healing. Adv. Healthc. Mater..

[134] Zhu J., Li F.X., Wang X.L., Yu J.Y., Wu D.Q. (2018). Hyaluronic acid and polyethylene glycol hybrid hydrogel encapsulating nanogel with hemostasis and sustainable antibacterial property for wound healing. ACS Appl. Mater. Interfaces.

[135] Golafshan N., Rezahasani R., Esfahani M.T., Kharaziha M., Khorasani S.N. (2017). Nanohybrid hydrogels of laponite: PVA-Alginate as a potential wound healing material. Carbohydr. Polym..

[136] Zhao P.X., Feng Y., Zhou Y.Q., Tan C.Y., Liu M.X. (2023). Gold@Halloysite nanotubes-chitin composite hydrogel with antibacterial and hemostatic activity for wound healing. Bioact. Mater..

[137] Pillai N.S.M., Eswar K., Amirthalingam S., Mony U., Varma P.K., Jayakumar R. (2019). Injectable nano whitlockite incorporated chitosan hydrogel for effective hemostasis. ACS Appl. Bio Mater..

[138] Pan G.X., Li F.H., He S.H., Li W.D., Wu Q.M., He J.J., Ruan R.J., Xiao Z.X., Zhang J., Yang H.H. (2022). Mussel- and barnacle cement proteins-inspired dual-bionic bioadhesive with repeatable wet-tissue adhesion, multimodal self-gealing, and antibacterial capability for nonpressing hemostasis and promoted wound healing. Adv. Funct. Mater..

[139] Fan X.M., Wang S.B., Fang Y., Li P.Y., Zhou W.K., Wang Z.C., Chen M.F., Liu H.Q. (2020). Tough polyacrylamide-tannic acid-kaolin adhesive hydrogels for quick hemostatic application. Mater. Sci. Eng. C.

[140] Gaharwar A.K., Avery R.K., Assmann A., Paul A., McKinley G.H., Khademhosseini A., Olsen B.D. (2014). Shear-Thinning Nanocomposite Hydrogels for the Treatment of Hemorrhage. ACS Nano.

[141] Yuan Y.C., Ding L.P., Chen Y., Chen G.Q., Zhao T.B., Yu Y.L. (2022). Nano-silver functionalized polysaccharides as a platform for wound dressings: A review. Int. J. Biol. Macromol..

[142] Zhao B.N., Zhang Y.Z., Li D.D., Mo X.M., Pan J.F. (2022). Hofmeister effect-enhanced gelatin/oxidized dextran hydrogels with improved mechanical properties and biocompatibility for wound healing. Acta Biomater..

[143] Arno M.C., Inam M., Weems A.C., Li Z.H., Binch A.L.A., Platt C.I., Richardson S.M., Hoyland J.A., Dove A.P., O'Reilly R.K. (2020). Exploiting the role of nanoparticle shape in enhancing hydrogel adhesive and mechanical properties. Nat. Commun..

[144] Zhang M.Y., Lin P.L., Song X.F., Chen K., Yang Y.X., Xu Y.L., Zhang Q., Wu Y.S., Zhang Y.F., Cheng Y.L. (2022). Injectable and self-healing hydrogels with tissue adhesiveness and antibacterial activity as wound dressings for infected wound healing. J. Polym. Sci..

[145] Lu J.W., Fan X.K., Hu J.W., Li J., Rong J.J., Wang W.J., Chen Y., Liu W.Y., Chen J., Chen Y. (2023). Construction and function of robust and moist bilayer chitosan-based hydrogel wound dressing. Mater. Des..

[146] Xie T., Ding J., Han X.X., Jia H.Z., Yang Y., Liang S., Wang W.X., Liu W.G., Wang W. (2020). Wound dressing change facilitated by spraying zinc ions. Mater. Horizons.

[147] Qu J.H., Li J., Zhu W.P., Xu Y.F., Zhou S.M., Yang Y.Y., Qian X.H. (2022). Hybrid nanocomposite multinetwork hydrogel containing magnesium hydroxide nanoparticles with enhanced antibacterial activity for wound dressing applications. Polymer.

[148] Wang S.G., Shi F.W.K., Yuan J.F., Sun W.L., Yang J.T., Chen Y.X., Zhang D., Che L.B. (2022). Osteichthyes skin-inspired tough and sticky composite hydrogels for dynamic adhesive dressings. Compos. B Eng..

[149] Hosseini S.F., Rezaei M., Zandi M., Farahmandghavi F. (2016). Development of bioactive fish gelatin/chitosan nanoparticles composite films with antimicrobial properties. Food Chem..

[150] Liu W.Z., Huang F., Wang Y.J., Zou T., Zheng J.S., Lin Z. (2011). Recycling Mg(OH)_2_ nanoadsorbent during treating the low concentration of Cr^VI^. Environ. Sci. Technol..

[151] Qiao Z., Parks J., Choi P., Ji H.F. (2019). Applications of Highly Stretchable and Tough Hydrogels. Polymers.

[152] Zheng Y.Q., Zhang K.Y., Yao Y.M., Li X.R., Yu J.Y., Ding B. (2021). Bioinspired sequentially crosslinked nanofibrous hydrogels with robust adhesive and stretchable capability for joint wound dressing. Compos. Commun..

[153] Hu T., Wu G.P., Bu H.T., Zhang H.Y., Li W.X., Song K., Jiang G.B. (2022). An injectable; adhesive, and self-healable composite hydrogel wound dressing with excellent antibacterial activity. Chem. Eng. J..

[154] Yang J., Li S.K., Yan L.L., Huo D.M., Wang F.C. (2010). Dynamic compressive properties and failure mechanism of glass fiber reinforced silica hydrogel. Mater. Sci. Eng. A.

[155] Shi S., Lan X., Feng J.X., Ni Y.S., Zhu M.Q., Sun J., Wang J.L. (2022). Hydrogel loading 2D montmorillonite exfoliated by anti-inflammatory *Lycium barbarum* L. polysaccharides for advanced wound dressing. Int. J. Biol. Macromol..

[156] Tao S.W., Zhu L., Xu N. (2021). Exosomes from Stem Cells Loaded by Hydrogel in Skin Wound Repair. Chin. J. Biochem. Mol. Biol..

[157] Wang L.L., Janes M.E., Kumbhojkar N., Kapate N., Clegg J.R., Prakash S., Heavey M.K., Zhao Z., Anselmo A.C., Mitragotri S. (2021). Cell therapies in the clinic. Bioeng. Transl. Med..

[158] (2022). KBV Research, Global Hydrogel Dressing Market Size, Share & Industry Trends Analysis Report By Application, By End-use, By Product, By Regional Outlook and Forecast, 2022–2028. https://www.reportlinker.com/p06364533/Global-Hydrogel-Dressing-Market-Size-Share-Industry-Trends-Analysis-Report-By-Application-By-End-use-By-Product-By-Regional-Outlook-and-Forecast.html?utm_source=GNW.

